# Cdc42 Interaction with N-WASP and Toca-1 Regulates Membrane Tubulation, Vesicle Formation and Vesicle Motility: Implications for Endocytosis

**DOI:** 10.1371/journal.pone.0012153

**Published:** 2010-08-13

**Authors:** Wenyu Bu, Kim Buay Lim, Yuan Hong Yu, Ai Mei Chou, Thankiah Sudhaharan, Sohail Ahmed

**Affiliations:** Neural Stem Cell Laboratory, Institute of Medical Biology, Singapore, Singapore; University of Birmingham, United Kingdom

## Abstract

Transducer of Cdc42-dependent actin assembly (Toca-1) consists of an F-BAR domain, a Cdc42 binding site and an SH3 domain. Toca-1 interacts with N-WASP, an activator of actin nucleation that binds Cdc42. Cdc42 may play an important role in regulating Toca-1 and N-WASP functions. We report here that the cellular expression of Toca-1 and N-WASP induces membrane tubulation and the formation of motile vesicles. Marker and uptake analysis suggests that the tubules and vesicles are associated with clathrin-mediated endocytosis. Forster resonance energy transfer (FRET) and Fluorescence Lifetime Imaging Microscopy (FLIM) analysis shows that Cdc42, N-WASP and Toca-1 form a trimer complex on the membrane tubules and vesicles and that Cdc42 interaction with N-WASP is critical for complex formation. Modulation of Cdc42 interaction with Toca-1 and/or N-WASP affects membrane tubulation, vesicle formation and vesicle motility. Thus Cdc42 may influence endocytic membrane trafficking by regulating the formation and activity of the Toca-1/N-WASP complex.

## Introduction

The uptake of nutrients and proteins and recycling of receptors, the process of endocytosis, represents a fundamental aspect of the cell biology of all eukaryotic cells. In particular, defects in these pathways have been linked to a wide range of disease states from cancer to neurodegeneration [1]. The small GTPases of the Ras superfamily are well known to have roles in endocytosis [2], [3]. For example, RhoB and RhoD regulate endosomal trafficking in co-operation with mDia1 and Src kinase [4]. Cdc42, a protein directly connected with cell migration and cell polarity, has also been linked to endocytosis [5]–[7]. The mechanism(s) by which Cdc42 may regulate endocytosis and/or membrane trafficking is unclear.

A theme emerging from recent work on FCH-Bin-Amphiphysin-Rsv (F-BAR) domain proteins and endocytosis is that there is a requirement to couple membrane remodeling with microfilament and microtubule dynamics [8]. The family of BAR domain proteins (classical BAR, F-BAR and Inverse-BAR [I-BAR]; see [9]–[11], for recent reviews) play important roles in remodeling membranes. Current models suggest that BAR domain proteins form dimers (and oligomers) and by so doing induce curvature on lipids allowing restructuring of membranes [11]–[13].

For the present study, of particular importance are the proteins of the Toca family (Toca-1, Cdc42 interacting protein 4 [CIP4; Toca-2] and formin binding protein 17 [FBP17; Toca-3]). These proteins share overall domain sequence and structure similarity; F-BAR domain, Cdc42 binding site, and Src homology 3 (SH3) domain. Further, the SH3 domain of these proteins binds to neuronal-Wiskott Aldrich syndrome protein (N-WASP) and dynamin [13]–[15]. FBP17 is the most well studied member of the family and is shown to induce tubular membrane invaginations and participate in endocytosis [16]. Toca-1 was identified in a protein purification scheme using Cdc42 activated actin polymerization from *Xenopus* cell extracts. Protein-protein interaction and reconstitution assays using Toca-1 have shown that it forms a complex with N-WASP through SH3 domain-polyproline rich domain interactions and that Cdc42 can activate the Toca-1/N-WASP complex to nucleate actin filaments *via* the Arp2/3 complex [17]. *In vitro* data suggest that Toca-1 and Cdc42 regulate N-WASP-Arp2/3 interaction and actin polymerization by relieving inhibitory intramolecular interactions of the WA (W [Verprolin, Cofilin] and Acidic region; Arp2/3 interacting) domain. Most recently, Toca-1 has been shown to induce filopodia formation in an N-WASP dependent manner [18]. Further, Toca-1 localizes to endocytic vesicles thus potentially linking the processes of filopodia formation and endocytosis [18].

Here we aim to understand the importance of Cdc42 in regulating the function of Toca-1 and N-WASP and have investigated the cellular function of Cdc42-N-WASP-Toca-1 complex. FBP17 and CIP4 and their F-BAR domains alone tubulate membranes *in vivo* [13], [16], [19]. In contrast, the Fes CIP4 homology domain (FCH; partial F-BAR domain), F-BAR domain, or full-length Toca-1 protein did not induce membrane tubulation. Coexpression of Toca-1 with N-WASP does tubulate membranes and induces the formation of motile membrane vesicles. Marker and uptake analysis suggests that the tubules and vesicles are associated with clathrin-mediated endocytosis. FRET shows that Cdc42, N-WASP and Toca-1 form a trimer complex on the membrane tubules and vesicles and that Cdc42 interaction with N-WASP is critical for complex formation. Modulation (by using mutants and inhibitors) of Cdc42 interaction with Toca-1 and/or N-WASP affects membrane tubulation, vesicle formation and vesicle motility. Thus Cdc42 may influence endocytic membrane trafficking by regulating the formation and activity of the Toca-1/N-WASP complex.

## Materials and Methods

### Materials

GFP-clathrin was from Prof James Keen [20]. Caveolin-1-mRFP was from Prof Richard E. Pagano (Addgene plasmid 12681). CFP-Akt-PH, CFP-PLCδ-PH and GFP-Btk-PH were provided by Dr Koichi Okumura (NUS, Singapore). GFP-Rab5 was provided by Prof Cecilia Bucci. All cell culture reagents are from Invitrogen. Cdc42 interacting domain of WASP (amino acid residues 215–295) was a kind gift from David B. Sacks (Harvard Medical School, Boston). Cyt. D (Cytochalasin D) was purchased from Calbiochem.

### Mammalian cell culture and transfection

CHO cells (ATCC, U.S.A) were maintained in F-12 Nutrient mixture (Kaighn's modification) media containing 10% Fetal Bovine Serum Qualified (FBS) and 1% antibiotics (penicillin and streptomycin) in a humidified 37°C incubator with 5% CO_2_/95% air. CHO cells were transfected using Fugene6 (Roche) according to the manufacturer's manual. HeLa cells (ATCC, U.S.A) were maintained in DMEM medium supplemented with 10% FBS and 1% antibiotics (penicillin/streptomycin) in a humidified 37°C incubator with 5% CO_2_/95% air. HeLa cells were transfected using Fugene6 (Roche) according to the manufacturer's manual.

### Immunofluorescence

The cells were fixed in 4% paraformaldyhyde for 15 min followed by three washes with PBS for five min each. Then the cells were permeabilized in 0.2% Triton X-100 for 5 min. After three washes with PBS, the cells were blocked in 5% normal goat serum for 30 min. The cells then were washed and incubated in PBS diluted primary antibody (anti-Rab5 (1∶50), anti-Rab7 (1∶50) and anti-Lamp1 (1∶50), all from Santa Cruz) at 4°C overnight. After three washings, the cells were incubated with Alexa488, Alexa594, or Cy5 conjugated secondary antibody. Finally the cells were washed three times and mounted onto the glass slides using Hydromount (National Diagnostics).

### Subcloning

Human Toca-1 was amplified by PCR and subcloned from pCS+ vector into pXJ40- mRFP vector between HindIII and NotI site. FCH (amino acid residues 2–94) and F-BAR (amino acid residues 2–293) were amplified by PCR using Myc-Toca-1/pCS+ as a template and was subcloned into pXJ40-GFP between BamH1 and XhoI site.

### Site directed mutagenesis

Myc-Toca-1W518K, mRFP-Toca-1W518K, mRFP-Toca-1K33QR35Q, mRFP-Toca-1K51QK52Q, mRFP-Toca-1R112QK113Q, mRFP-Toca-1MGD383-385IST and GFP-N-WASPH208D were generated by mutated primer pairs using site-directed mutagenesis kit (Stratagene). The mutations were confirmed by DNA sequencing. For details of mutagenesis methods see [18]. The list of mutants used in this study was shown in Table S1.

### FRET measurement

FRET was measured by (AP)-acceptor photobleaching method [21], [22] by making appropriate settings in a Zeiss inverted laser scanning confocal microscope (LSM510) with the objective of C-Apochromat 63×/1.2 water immersion objective. The fusion proteins of GFP/mRFP were excited using 488 and 561 nm laser line as excitation source, by selecting 405/488/561 nm dichoric mirror and 490, 565 nm secondary dichoric mirrors for GFP and mRFP emission respectively. The emission was monitored by selecting GFP (BP 505–550 nm) and Red (Long pass 575 nm) emission filters to record the fluorescence intensity. Region of interest (ROI) was selected and photobleached using 70% of 561 nm laser power by selecting 50 iterations. Bleaching was performed following pre scan images. The increase in GFP fluorescence intensity followed by mRFP bleaching was measured as FRET. FRET efficiency was calculated using the change in background subtracted fluorescence intensity as 100×[(post- bleach intensity)−(pre-bleach intensity)/(post- bleach intensity)].

In order to verify the increase in GFP intensity due to any possible artifact we obtained the Pearson product moment correlation coefficient r, a dimensionless index that ranges from −1.0 to 1.0 inclusive and reflects the extend of a linear relationship between the two fluorescence intensity data of GFP and mRFP while bleaching. In our case we expect −1.0 as the perfect fitting of the linear relation. However we selected the range of −0.7 to −1.0 as the best range of index.
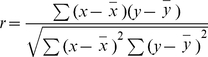
Where x and y are the sample means average (array1, GFP intensity) and average (array2, mRFP intensity). For complete details of AP-FRET method see [22].

### Fluoresence Recovery After Photobeaching (FRAP)

FRAP was performed on a Zeiss inverted LSM510 with C-Apochromat 63×/1.2 water immersion objective and a pinhole size 1 airy unit. The temperature was maintained at 37°C in a humidified atmosphere with 5% CO_2_. GFP tagged proteins were bleached in a defined rectangular region using a 488 nm laser line at 100% power and 25 iterations. The fluorescence recovery was recorded by a 488 nm laser line at 5% laser power every 1 second. The fluorescence intensity of the background, the whole cell and the bleached region were recorded before bleaching, just after bleaching and during the recovery. In the analysis of the FRAP data, the background fluorescence intensity was subtracted and the fluorescence intensity was normalized against fluorescence intensity of whole cell in order to correct the effect of the focal plane drifting and the photobleaching in the imaging process. Then each data point was normalized to the fluorescence intensity before bleaching to get the relative fluorescence intensity in a region of interest (ROI). The relative intensity (RI) was calculated using the following equation: RI = [It(0)/ROI(0)]/[ROI(t)/It(t)], It(0), whole cell intensity before bleaching; ROI(0), intensity of bleached region before bleaching; It(t), whole cell intensity at a certain time point; ROI(t), intensity of bleached region at a certain time point. T_1/2_ was calculated as the time required recovering to 50% of the fluorescence before bleaching.

### FLIM

FLIM experiments were performed with the LIFA system (Lambert Instruments, The Netherlands) on an inverted wide-field fluorescence microscope (Olympus IX71, Center Valley, PA) with 60×/1.35 oil immersion objective. Fluorescence lifetime was measured using software provided by Lambert Instruments. GFP was excited by a sinusoidally modulated 4 mW 470 nm light-emitting diode at 40 MHz. The GFP filter set was used for excitation and emission signals. The emission was collected by an intensified CCD camera. Fluorescein isothiocyanate was used as a standard lifetime reference of 4 ns. 12 phase and modulation shifted images were taken, fitted with a sinus function and used for the calculation of lifetime (for further details see [18]). The lifetimes from 50 ROIs were taken from different cells are averaged to give the average lifetime and standard deviation. The experiments were repeated three times and data from a single representative experiment are shown. The lifetime (ns) is shown in pseudo colours.

### Confocal microscopy and time-lapse acquisition

Immunofluorescence was examined on an Olympus FV1000 confocal microscope (Olympus, Japan) using a 63×/1.4 oil immersion objective with a pinhole at airy 1. GFP tagged proteins were excited by a 488 nm laser line and the emission was selected using a band-pass filter between 500–550 nm. mRFP tagged proteins were excited by a 561 nm laser line and the emission was selected using a band-pass filter between 580–680 nm. The images were taken at a speed 2 µs/pixel sequentially at a size 512×512. The time lapse images were taken at 10 sec interval in a humidified chamber at 37°C with 5% CO_2_.

### Total Internal Reflection Fluorescence Microscopy (TIRFM)

To examine the vesicle dynamics at the cell surface, TIRFM was performed on an inverted Olympus FV TIRF microscope in a humidified atmosphere with 5% CO_2_. Plan-Apochromat 63×/1.45 oil-immersion objective was used and 488 nm/561 nm laser line were used to excite GFP and mRFP sequentially. The penetration depth was set at around 100 nm. Light emitted by the fluorophores was detected by a CoolSnap HQ^2^ camera (Photometrics, USA). Metamorph software controls the multidimensional time-lapse acquisition. The exposure time was 200 ms and total 360 frames were taken with 5 sec interval. The stack files were processed using Metamorph to generate movie files.

### Uptake assays

Quantification of fluorescence intensities was performed using Metamorph software (Universal Imaging, PA, USA), as previously describe in [18]. Briefly, fluorescence intensities were measured by thresholding and outlining whole individual cells, followed by determination of integrated fluorescence intensities, which were then normalized to cell area. Average values were expressed as a ratio relative to control cells without transfection.

*(a) Transferrin uptake assay.* The cells were serum starved for 45 min and FITC labeled transferrin (Molecular Probes) was added to the cell at a final concentration 25 µg/ml. After incubation for 10 min at 37°C, the cells were washed twice with Dulbecco's PBS containing 1 mM CaCl_2_ and 0.5 mM MgCl_2_ (D-PBS; Invitrogen) at 4°C. To reduce cell surface labeling, subsequently the cells were incubated in 50 µM of deferoxamine (Sigma), an iron chelator, in 150 mM NaCl, 2 mM CaCl_2_, 25 mM sodium acetate/acetic acid, pH 4.5, for 10 min [23], and re-equilibrated with two additional washes with ice-cold D-PBS. The coverslip was mounted onto glass slide using hydromount and examined with Olympus FV1000 confocal microscopy.

*(b) Cholera toxin B uptake assay.* Cells grown on coverslips to 70–80% confluency were rinsed twice with D-PBS and serum-starved for 1 hr in serum-free medium. Cells were incubated with 5 µg/ml Alexa 488-cholera toxin B (Molecular Probes) in serum-free DMEM for 30 min at 37°C and 5% CO_2_. Subsequently, coverslips were placed on ice and rinsed twice with ice-cold D-PBS and mounted onto glass slide using hydromount and examined by confocal microscopy.

*(c) Dextran uptake assay.* Cells were serum starved for 1 hr and dextran was added into the culture medium at a final concentration of 1 mg/ml. The cells were then incubated in 37°C for 30 min. Subsequently the cells were washed twice with ice-cold D-PBS and mounted using hydromount.

### Quantification of tubulation, vesicle number and motility

*(a) Tubule and vesicle index.* Cells were divided into eight sectors and each sector was scored for the presence of vesicles or tubules. Each sector accounts for 12.5% of total morphological activity. The eight sector values for each cell were then added to give % vesicle/tubule index per cell. *(b) Vesicle number.* Vesicle number was analyzed by selecting fixed areas within each cell and counting the number of vesicles present. Vesicle numbers were then normalized to the non-transfected cell. The experiments were repeated 2–3 times, with n = 20, as the mean +/− S.D. (standard deviation) and a representative experiment is presented. *(c) Vesicle motility.* Vesicle motility was tracked using Metamorph software.

### Statistical analysis

In general, readings were obtained from at least 3 independent experiments, and expressed as the mean +/− S.D.. Experimental data were analyzed by Student's t-test. Difference was significant when p<0.05. (* stands for p<0.05; ** for p<0.01; *** for p<0.001).

## Results

### Toca-1/N-WASP interaction induces the formation of dynamic membrane tubules and vesicles

**Figure 1:**
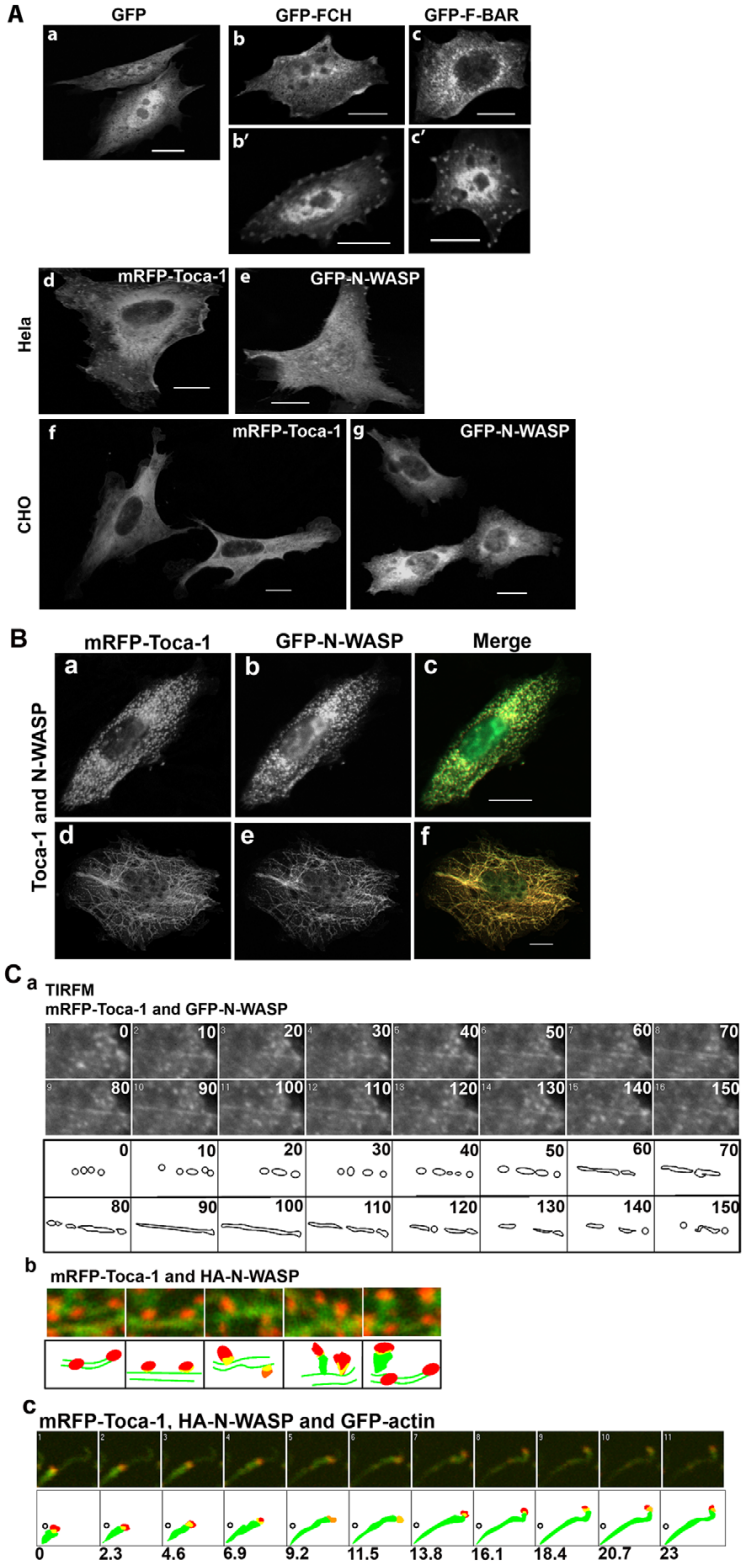
Characteristics of Toca-1/N-WASP induced membrane tubulation, vesicle formation and motility. (A) Cells were transfected with cDNA encoding; GFP alone (panel a), GFP-FCH domain (panels b,b′), GFP-F-BAR domain (panels c,c′). mRFP-Toca-1 or GFP-N-WASP in HeLa cells (panels d,e). mRFP-Toca-1 or GFP-N-WASP in CHO cells (panels f,g). Toca-1 and N-WASP expressed individually induce filopodia formation as described in [18] but not tubules or vesicles. (B–C) CHO cells were transfected with mRFP-Toca-1 and GFP-N-WASP (B, C part a) or with mRFP-Toca-1/HA-N-WASP and GFP-actin (C, parts b and c) as described in the Material and Methods section. (B) Confocal image of cells expressing mRFP-Toca-1-GFP-N-WASP. mRFP-Toca-1 (a and d), GFP-N-WASP (b and e) and the merge (c and f), green – N-WASP and red – Toca-1. Upper panels show vesicles (a–c) and lower panels (d–f) tubules. (C). TIRF live cell microscopy was used to follow the dynamics of tubule and membrane vesicle formation at the membrane (a). A time lapse sequence of mRFP-Toca-1 is shown with time in sec. The lower panels show a schematic of the time-lapse sequence illustrating how vesicles align to form tubules which then disassemble to give vesicles. (b) The process of vesicle formation from tubules is shown with mRFP-Toca-1 and GFP-actin as the two labels being followed. N-WASP is present but silent in HA tagged form. (c) mRFP-Toca/HA-N-WASP and GFP-actin were used to follow actin polymerization during vesicle motility. One vesicle was selected for analysis. Background signals were removed to allow clear visualization of the relationship between mRFP-Toca-1 and GFP-actin. Similar results were obtained with mRFP-N-WASP/myc-Toca-1 and GFP-actin. Bar = 10 µm. Movies S1, S2, S3 illustrate the dynamics of the Toca-1/N-WASP phenotype (see Suppl. data).

Tubulation reflects F-BAR domain mediated membrane deformation activity. In vitro tubulation can be seen as the deformation of lipid vesicles. FBP17 and CIP4 and their F-BAR domains alone tubulate membranes *in vivo* [13], [16], [19]. In contrast, the FCH (partial F-BAR domain), F-BAR domain, or full-length Toca-1 protein did not induce membrane tubulation (Fig. 1A). This suggests that the F-BAR domain of Toca-1 is more tightly regulated than that of CIP4 and FBP17. However, coexpression of Toca-1 with N-WASP was sufficient to induce the formation of membrane tubules and vesicles in CHO cells (Fig. 1B) and HeLa, COS7 and N1E115 cells (Fig. S1). Using time-lapse TIRFM we examined the dynamics of the processes of membrane tubulation and vesicle formation. In cells that contained few tubules, the vesicles were seen to align and form tubules. This sequence was reversible as tubules could be seen to disintegrate and give rise to vesicles (Fig. 1C, a; see movies S1 and S2). Thus tubule formation is a result of dynamic membrane deformation in the absence of vesicle scission. Using GFP-actin and mRFP-Toca-1/HA-N-WASP we were able to observe vesicle formation from tubules and follow actin dynamics (Fig. 1C, b). Vesicle formation involved scission from the tubule and was possibly driven by actin polymerization. The vesicles themselves were motile with a speed of approx. 60 nm/sec (Fig. 1C, c). Both vesicle formation and motility were associated with an actin comet emerging behind the direction of movement (Fig. 1C; see movie S3) reminiscent of viral/bacterial movement in mammalian cells (e.g. [24], [25]).

**Figure 2:**
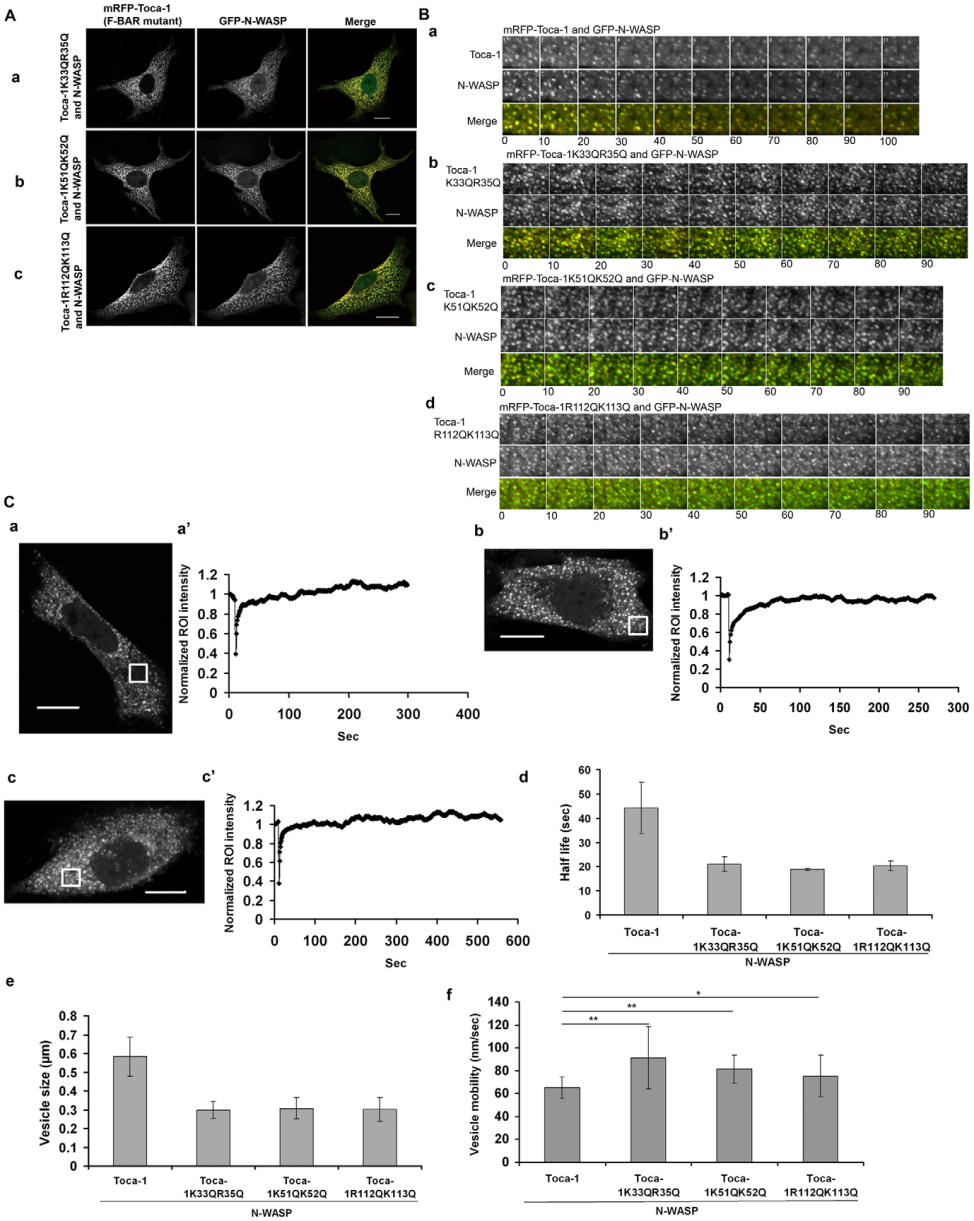
Effect of Toca-1 F-BAR mutants on Toca-1/N-WASP phenotypes. Cells were transfected with mRFP-Toca-1 F-BAR mutants with N-WASP as outlined in the figures. (A). Phenotypes of Toca-1 F-BAR domain mutant/N-WASP combinations. (B) Motility of wild-type vesicles and Toca-1 F-BAR domain mutant generated vesicles. (C) FRAP analysis of Toca-1F-BAR domain mutant generated vesicles (a–d). Effect of Toca-1 F-BAR domain mutants on vesicle size (e) and motility (f). Bar = 10 µm. For statistical analysis numbers are averages +/− S. D., with n = 5–15, from 2-3 experiments.

Toca-1 F-BAR domain mutants failed to form tubules when expressed with N-WASP suggesting that F-BAR domain activity is essential for tubule formation (Fig. 2A). Vesicles are still formed when Toca-1 F-BAR domain mutants were coexpressed with N-WASP. This may be explained by the Toca-1mutant/N-WASP complex still being competent to promote scission of preexisting deformed membranes. We then analyzed these vesicles in detail. The vesicles formed by F-BAR domain mutated Toca-1 and N-WASP are more motile and smaller in size than vesicles formed by wild type Toca-1 and N-WASP (Fig. 2B and C, e–f). Consistent with this, the vesicles formed by Toca-1 F-BAR domain mutants recover faster than the controls in FRAP experiments (Fig. 2C, a–d).

### Characteristics of Toca-1/N-WASP induced membrane tubules and vesicles

To understand the function of the tubules and vesicles formed by Toca-1/N-WASP expression we next carried out a marker analysis. We found that the tubules were colocalised with the plasma membrane target (PMT) sequence [26], [27] (Table 1 and Fig S2). The vesicles induced by Toca-1/N-WASP were positive for Rab5 and Rab7 but not Lamp1 (Table 1 and Fig. S3A). In contrast, the tubules, did not stain positive for Rab5, Rab7 or Lamp1. This suggests that tubules are not linked directly to endosomal membranes, but rather to the plasma membrane. In addition, we observed the tubule formation under TIRFM which can only image approx 100 nm from the coverslip. Toca-1 family members, FBP17 and CIP4, generate tubules that colocalise with the plasma membrane [14]–[16]. Taken together, these results strongly support the view that tubules are derived from the plasma membrane and not the endosomal membrane.

**Table 1:** Colocalization analysis of Toca-1 and N-WASP expressing cells.

Vesicle		
Marker	Colocalization	Pearson CC ± S.D.
GFP-actin	**	0.55±0.09
GFP-clathrin	***	0.73±0.04
Rab5	**	0.67±0.10
Rab7	**	0.51±0.12
Lamp-1	-	0.16±0.08
RFP-caveolin	-	0.10±0.08
CFP-PLCδ-PH	***	0.71±0.04
CFP-Akt-PH	***	0.55±0.06
GFP-Btk-PH	***	0.74±0.05
Phalloidin (F-actin)	**	0.53±0.11

Tubule		
Marker	Colocalization	
GFP-actin	***	0.72±0.05
CFP-PLCδ-PH	***	0.70±0.06
CFP-Akt-PH	***	0.60±0.05
GFP-Btk-PH	***	0.75±0.12
Phalloidin (F-actin)	***	0.75±0.05
GFP-clathrin	***	0.75±0.07
YFP-PMT	***	0.72±0.09

Cells were transfected with Toca-1 and N-WASP and left for 24–36 hr. After fixation cells were incubated with primary antibody followed by secondary antibody for markers of membrane trafficking pathways as described in the Material and methods section. PH domains were visualized with GFP, or CFP. Olympus FV1000 confocal microscope software was used for colocalization analysis. Where Pearson colocalization coefficient (CC) of; <0.25 indicates no colocalization (-), 0.25–0.5 indicates potential colocalization (*), 0.5–0.7 indicates some colocalization (**) and >0.7, indicates high colocalization (***). The CC is an average +/− S. D., with n = 8–10, from 3 experiments.

Clathrin colocalized with Toca-1 in the membrane tubules and vesicles but caveolin did not (Table 1 and Fig. S3C and S3D). PH (pleckstrin homology) domain probes colocalized in the membrane tubules and vesicles suggesting the involvement of phosphoinositides, and PIP_2_ (Phosphatidylinositol 4,5-bisphosphate) in particular (Table 1 and Fig. S3E).

We next examined which membrane uptake pathway was being affected by coexpression of Toca-1/N-WASP. Using transferrin, cholera toxin B and dextran uptake we found that only transferrin uptake was inhibited. The inhibition is likely due to high levels of tubule and vesicle formation perturbing the normal uptake process. Inhibition of transferrin uptake was dependent on the interaction of Toca-1/N-WASP as a SH3 domain mutant of Toca-1W518K, that does not interact with N-WASP [17], coexpressed with N-WASP, did not affect transferrin uptake. Further, expression of Toca-1 or N-WASP alone did not affect transferrin uptake significantly (Table 2 and Fig. S4). Taken together these data are consistent with a role of Toca-1/N-WASP in early clathrin-mediated endocytosis.

**Table 2:** Uptake assays of endocytic markers by Toca-1 and N-WASP expressing cells.

Endocytic pathway	Marker	Toca-1-N-WASP expression (% of control)
Clathrin-mediated endocytosis	Transferrin	50.2±11.6
Caveolin-medicated endocytosis	Cholera toxin B	103.2±8.7
Pinocytosis	Dextran	112.1±19.3

Cells were transfected with Toca-1 and N-WASP and left to express the proteins for 24–36 hr. Markers for uptake pathways were then added and uptake monitored for 10 min for transferrin and 30 min for dextran and cholera toxin B (For images see Fig. S4). The cells were then washed, fixed and examined by confocal microscopy. Quantification of the % uptake of different markers in cells transfected with Toca-1-N-WASP compared with control cells as described in Material and methods section. The % uptake is an average +/− S. D., with n = 10, from 3 experiments.

### Role of F-actin on Toca-1/N-WASP phenotypes and dynamics

**Figure 3:**
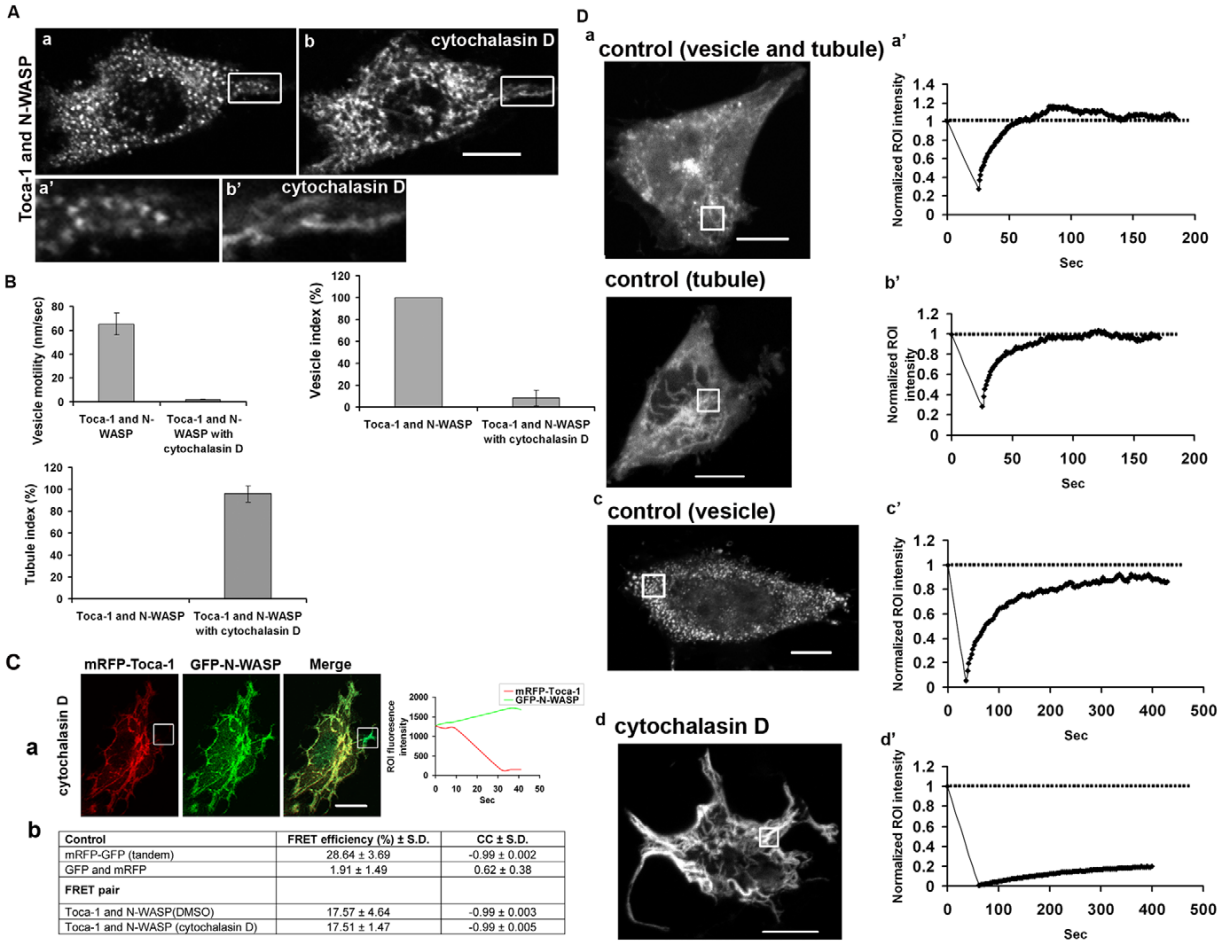
Effect of Cyt. D on the Toca-1/N-WASP phenotypes. (A). Cells were transfected with Toca-1 and N-WASP cDNA as described in the Material and methods sections. After 36 hr cells were chosen for either tubulation or membrane vesicle formation. Using time-lapse microscopy the change in phenotype was followed after addition of Cyt. D (4 µM) for 60 min. Panels show images at zero time (a, a′) and at 60 min (b, b′) of a representative cell. (a′b′) are enlargements of areas in panels a–b, respectively, showing (a′b′, Cyt. D) vesicle to tubule transitions. Bar = 10 µm. (B). Cells were then scored for presence of vesicles (vesicle index), tubules (tubule index) and vesicle motility as described in the Material and methods section. (C) Shows AP-FRET analysis of Toca-1/N-WASP in tubules or vesicles after Cyt. D treatment. (a) Images show typical cells used and the ROI. Green/red tracings show changes in intensity of the ROI before and after photobleaching. (b) A statistical analysis of FRET data with controls and FRET pairs. Bar = 10 µm. (D) FRAP analysis of the protein dynamics within the tubules/vesicles, with and without Cyt. D. Images show typical cells used and the graphs below show the bleach followed by the recovery profile. Time in sec. Bar = 10 µm. For statistical analysis numbers are averages +/− S. D., with n = 7–10, from 2–3 experiments.

Actin and actin filaments associate with the Toca-1-N-WASP induced membrane tubules and vesicles (Fig. S3B). To determine whether destruction of F-actin microfilaments affected the phenotype, Cyt. D was used (Fig. 3). Cyt. D inhibited vesicle formation (Fig. 3A) and the tubules were morphologically different (thicker and stabilized; see FRAP analysis below). Cyt. D did not affect the interaction of Toca-1 and N-WASP in membrane tubules and vesicles as seen by AP-FRET (Fig. 3C). Cyt. D inhibited vesicle motility (Fig. 3B).

To compare protein dynamics within membrane vesicles and tubules under the different experimental conditions FRAP was used. Cells were transfected with Toca-1 and N-WASP cDNA, allowed to express for 36 hr and their phenotypes were followed. Fluorescence was monitored for approx. 30 sec pre-bleach to obtain a baseline, bleaching carried out and then recovery monitored for approx. 300 sec (Fig. 3D). For Cyt. D treatment, FRAP analysis was carried 60 min after treatment. FRAP analysis was carried out in an ROI incorporating either membrane vesicles or tubules. Toca-1-N-WASP induced membrane tubules and vesicles had similar recovery rates of approx. 44 sec. Interestingly, Cyt. D treatment, which eliminated membrane vesicle formation but not tubules formation, prevented fluorescence recovery after photobleaching (Fig. 3D, panel d). This suggests that actin polymerization and or filaments are required for tubule dynamics and vesicle formation.

### Toca-1 and N-WASP interact in tubules and vesicles

**Figure 4:**
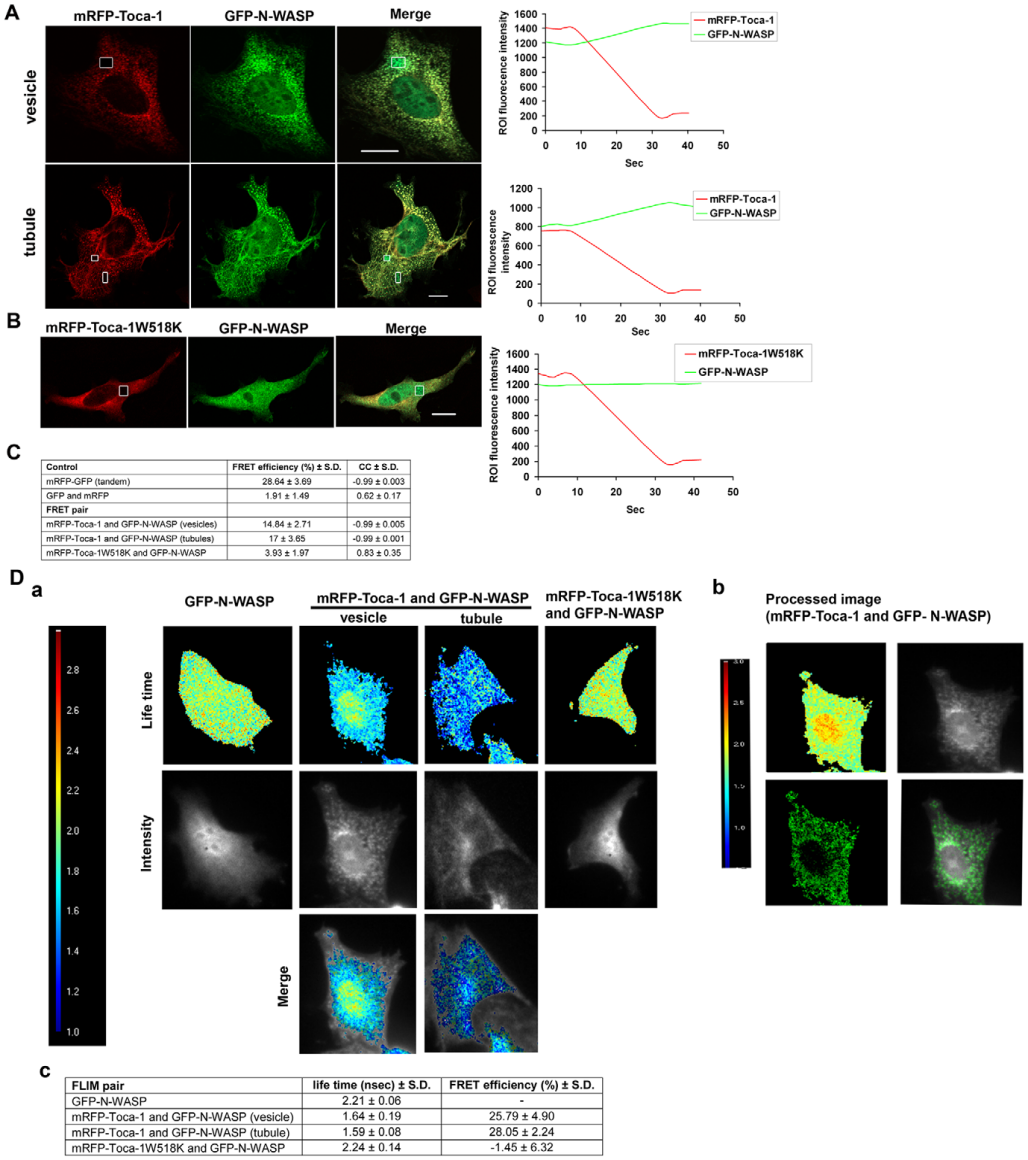
FRET and FLIM analysis of the Toca-1/N-WASP interaction. Cells were transfected with mRFP-Toca-1 and GFP-N-WASP cDNA and left to express mRFP/GFP for 36 hr as described in the Material and methods section. (A) Cells were selected for either a vesicle or tubule phenotype. ROIs focusing on these structures were chosen, as shown, and AP-FRET carried out as described in the Material and methods section. Green/red traces to the left of the cell images indicate mRFP/GFP fluorescence pre and post-bleach. The time course of these experiments is approx. 60 sec. (B) The Toca-1 SH3 domain mutant W518K (which is unable to bind N-WASP) was analyzed as shown for the wild-type in (A). (C) A summary of data obtained for controls as well as FRET pairs is shown. The CC shows the relationship between the mRFP and GFP signals during AP-FRET. GFP/mRFP FRET controls were as described in [22]. For the positive FRET scenario we expect high negative cross correlation between donor and acceptor signals. We define positive FRET when the FRET efficiency (FE) >3% and CC >−0.7. (D) Cells are analyzed by frequency-domain FLIM as described in the Material and methods section. (a) Lifetimes are colour coded (between 1.0–3.0 ns). Cells were chosen as for AP-FRET for the presence of either tubules or vesicles and then 12 phase shifted images captured and processed to generate a lifetime image. (b) These images were processed to demonstrate more clearly morphological structures as follows; the higher lifetime signals (the non-interacting signals) from the original FLIM image were masked in Photoshop and then (using Metamorph) the masked image was given a pseudo color and overlaid with intensity image. Lifetime images are shown with the intensity images below. The lifetimes of GFP-N-WASP within tubules and vesicles can be obtained using this analysis. (c) Summary of lifetimes obtained for controls, N-WASP alone, Toca-1/N-WASP and Toca-1W518K/N-WASP. Bar = 10 µm. For statistical analysis numbers are averages +/− S. D., with n = 7–15, from 2–3 experiments.

To determine whether the interaction of Toca-1 with N-WASP was important for the induction of membrane tubules and vesicles the W518K mutant of Toca-1 was used. Coexpression of Toca-1W518K with N-WASP did not induce membrane tubulation or the formation of vesicles (Fig. 4B). This result suggests that N-WASP plays an important role in control of the membrane tubulation activity of Toca-1.

To show conclusively that Toca-1/N-WASP interaction was important for the induction of membrane tubules and vesicles we carried out FRET experiments on these cellular structures. GFP-N-WASP and mRFP-Toca-1 were coexpressed and AP-FRET carried out on fixed samples. If N-WASP and Toca-1 are binding each other and the distance between GFP and mRFP is less than 10 nm FRET can occur. In this AP-FRET method the mRFP fluorescence is bleached and the GFP fluorescence followed pre and post bleach. In the FRET scenario GFP intensity will increase on mRFP bleaching. Further, in the FRET scenario the rates of change in GFP/mRFP fluorescence should be inversely correlated. The intensity changes following bleaching are expressed as a cross correlation coefficient (CC). In addition to these experiments we also carried out a number of controls. These include using free GFP/mRFP proteins as well as a tandem GFP-mRFP fusion protein. We define a positive FRET as %FE >3% and a CC between −0.7 to −1.0. Full details of this methodology can be found in [22]. Figure 4 shows examples of FRET experiments focusing on either membrane vesicles or tubules. Toca-1 and N-WASP not only colocalize in these membrane vesicles/tubules but also interact as seen by the positive FRET. In contrast, Toca-1W518K fails to FRET with N-WASP (Fig. 4A–C).

To confirm the AP-FRET results showing direct interaction of Toca-1 with N-WASP by an independent method we used frequency-domain FLIM. In this FRET method the lifetime of the donor is used as an indicator of protein-protein interaction. A positive FRET leading to decreased donor lifetime. The lifetime of GFP in cells is approximately 2.2 ns. If GFP is within 10 nm of an acceptor, such as mRFP, FRET can occur. With cytosolic GFP and mRFP FRET does not occur and coexpression does not affect GFP lifetimes (Fig. 4D). Using GFP-N-WASP and mRFP-Toca-1 expressing cells we measured the GFP lifetimes using the frequency domain method. With this method we were able to combine intensity measurements with lifetimes and obtain some spatial resolution of sites where Toca-1 interacted with N-WASP (Fig. 4D). Supporting the AP-FRET analysis Toca-1 interacted with N-WASP in vesicles with a lifetime of 1.64 ns and tubules with a lifetime of 1.59 ns (Fig. 4D). Similar FLIM data were obtained using time-domain measurements (data not shown).

### Dynamin is essential for vesicle formation but not for tubulation

**Figure 5:**
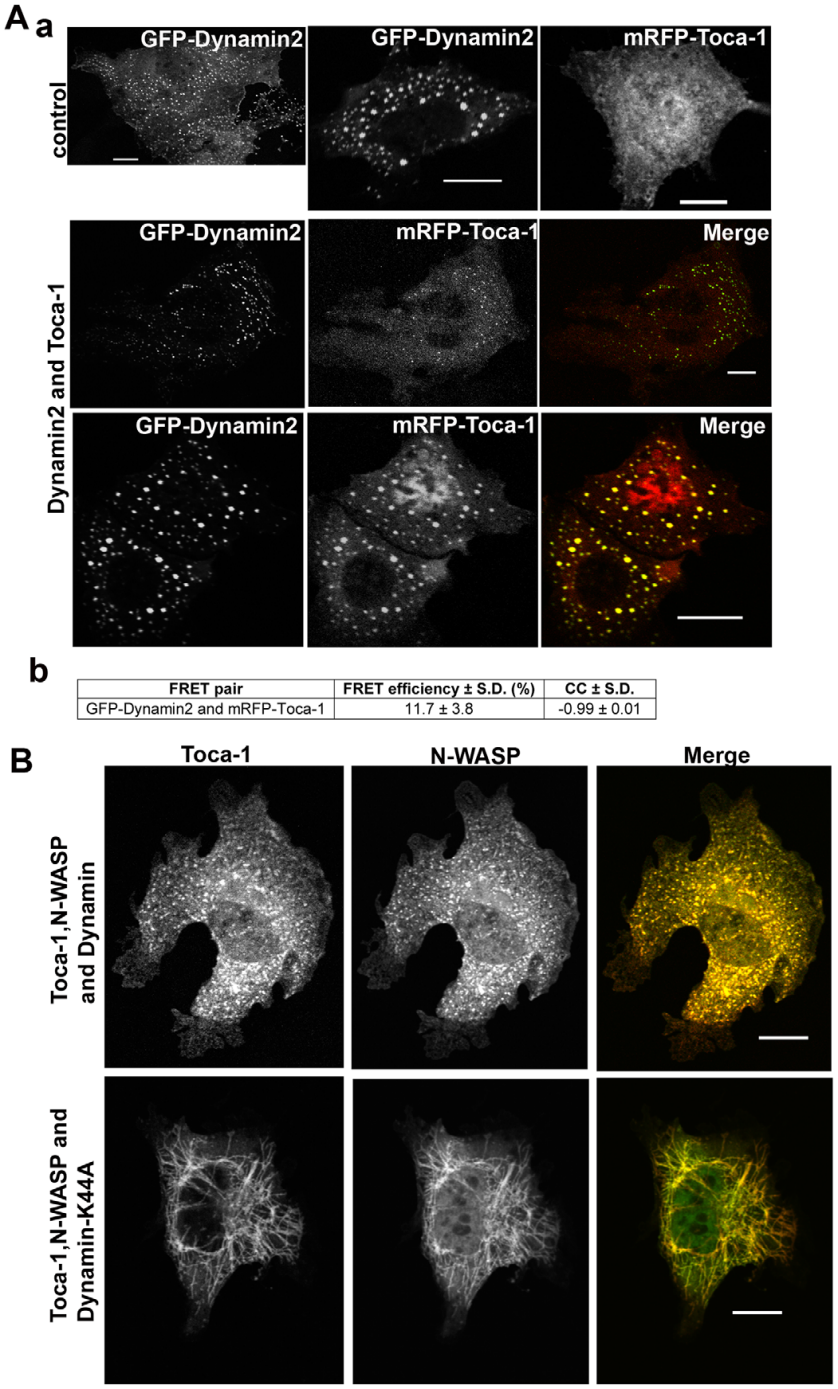
Dynamin function, Toca-1 interaction and phenotype. (A). Cells were transfected with mRFP-Toca-1 and either GFP-dynamin2 or GFP-dynamin1 cDNA (not shown) as described in the Material and methods. The top two panels show the cell phenotypes of individual protein expression. The lower two panels show the double expression with different level of protein expression. Bar = 10 µm. Numerical data are derived from AP-FRET analysis of mRFP-Toca-1/GFP-dynamin2. (B) mRFP-Toca-1, GFP-N-WASP and HA-dynamin transfections were carried as before (see figure 1 legend for details) and either dynamin1 WT (upper panel) or dynamin1-K44A (dominant negative) cDNA (lower panel). Three images for each combination is shown; Toca-1, N-WASP and merge (red – Toca-1; green – N-WASP). Single GFP/mRFP images are in black and white. Bar = 10 µm. For statistical analysis numbers are averages +/− S. D., with n = 8–10, from 2–3 experiments.

As Toca-1 has been reported to bind dynamin [15], we also examined the phenotype of Toca-1-dynamin coexpression (Fig. 5A). We expressed mRFP-Toca-1 with GFP-dynamin and both proteins were localized to membrane vesicles but tubules were not generated. Interestingly, the vesicles were not motile and were morphologically distinct from Toca-1/N-WASP vesicles and similar to the dynamin-carrying vesicles found in the absence of Toca-1 expression regardless of the protein expression level. Toca-1 was in a complex with dynamin in these membrane vesicles as seen by FRET analysis (Fig. 5A, b). We speculated that Toca-1/N-WASP mediated tubule formation arose because of limiting amounts of endogenous dynamin. To investigate this possibility further we used wild-type (WT) and K44A-dominant negative (DN)-dynamin. DN-dynamin prevented the formation of membrane vesicles and all transfected cells contained membrane tubules. In contrast, expression of WT-dynamin with Toca-1/N-WASP eliminated membrane tubules and cells were rich in membrane vesicles (Fig. 5B).

### Cdc42 regulates Toca-1/N-WASP complex formation

**Figure 6:**
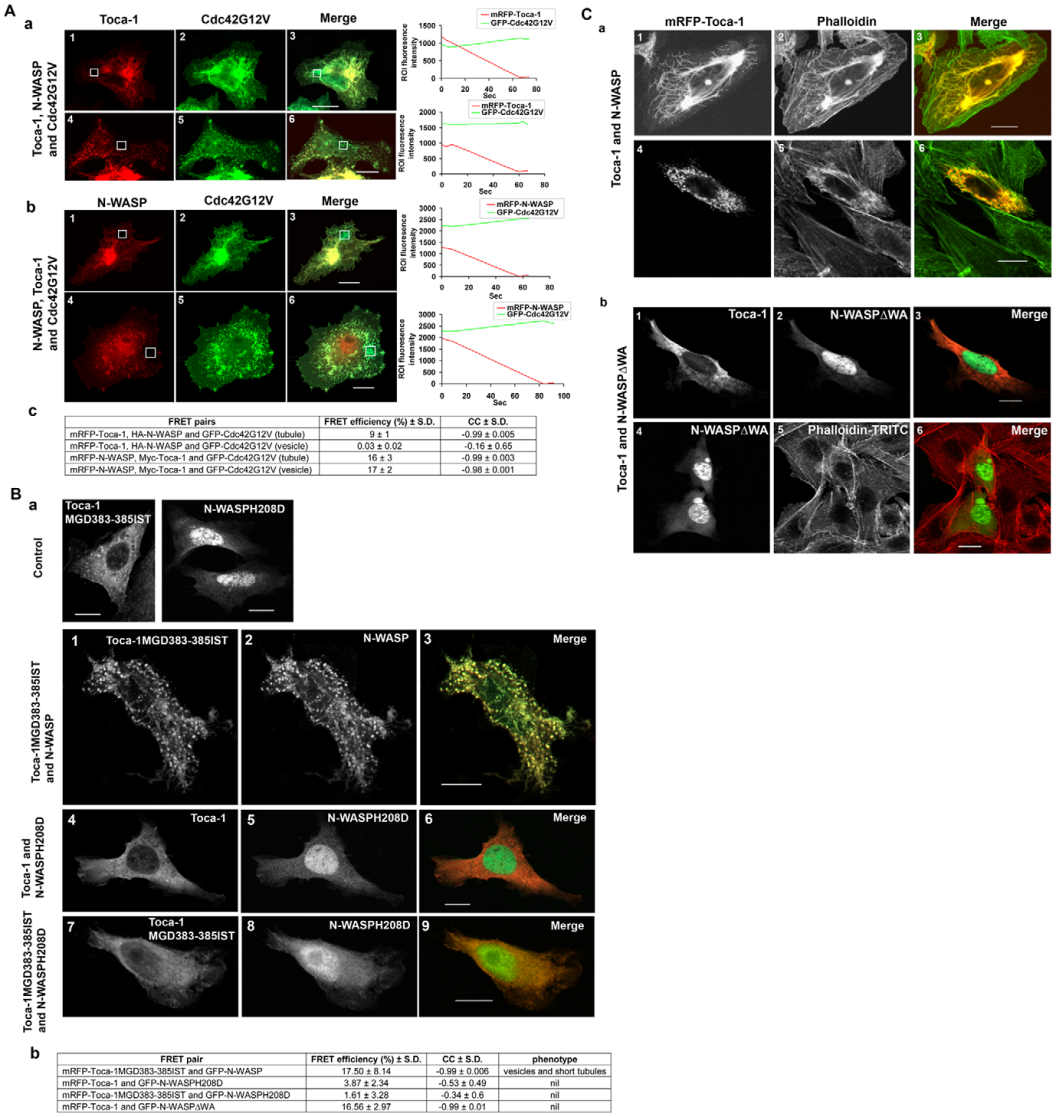
Cdc42, Toca-1 and N-WASP interactions and phenotypes. (A) Cells were transfected Toca-1, N-WASP and Cdc42G12V and allowed to express for 36 hours as described in the Material and methods section. To perform AP-FRET Cdc42G12V was GFP labeled and the other cDNA labeled with mRFP. The third cDNA encoded (myc or HA tagged) non-fluorescent protein. Cells with either tubules or vesicles were then chosen and AP-FRET performed on the ROI. (a) Cdc42-Toca-1 interaction (panels 1–3 tubules, panels 4–6 vesicles). (b) Cdc42-N-WASP interaction (panels 1–3 tubules, panels 4–6 vesicles). (c) A statistical analysis of the AP-FRET data. Traces on the right of the images represent intensity values of GFP and mRFP during pre and post bleach. (B) AP-FRET analysis of interactions between Toca-1 and N-WASP mutants. Cells were transfected with cDNAs endcoding GFP and mRFP fusions and allowed to express for 36 hr as described in the Material and methods. (a) Top two panels show single transfections, either Toca-1-MGD383-385IST mutant alone or N-WASPH208D mutant alone. The subsequent 9 panels show, in groups of three; Toca-1MGD383-385-IST/N-WASP (panels 1–3), Toca-1/N-WASPH208D (panels 4–6) and Toca-1MGD383-385IST/N-WASPH208D (panels 7–9). Left panels show Toca-1 and mutants, middle panels show N-WASP and mutants and right panels show merged images. (b) A statistical analysis of the AP-FRET experiments with associated phenotypes. Bar = 10 µm. (C) Effect Toca-1/N-WASP and Toca-1/N-WASPΔWA combinations on F-actin as stained by phalloidin. (a) Cells were transfected with cDNAs encoding GFP-N-WASP and mRFP-Toca-1 fusions or (b) mRFP-Toca-1/GFP-N-WASPΔWA as described in the Material and methods. F-actin was visualized with phalloidin. In (a) first series of three images is a cell with tubules. Second series of three shows a cell with vesicles. (b) First series of three images shows signals from Toca1, N-WASPΔWA and then the merge. Second series of three shows N-WASPΔWA, F-actin and then the merge. Bar = 10 µm. For statistical analysis numbers are averages +/− S. D., with n = 7–10, from 2–3 experiments.

We next investigated the role of Cdc42 in Toca-1/N-WASP complex formation and activity. First we determined whether Cdc42 did indeed interact directly with Toca-1 and/or N-WASP within the Toca-1/N-WASP complex in tubules and membrane vesicles using AP-FRET. These experiments involved using low level expression of GFP-Cdc42G12V (which did not significantly affect phenotype) in combination with either mRFP-Toca-1/HA-N-WASP or mRFP-N-WASP/myc-Toca-1. Cdc42G12V interacted with Toca-1 in tubules but not in vesicles (Fig. 6A). Cdc42 interacted with N-WASP in both tubules and membrane vesicles. Thus the Cdc42-N-WASP-Toca-1 complex does exist in both tubules and vesicles; however, Cdc42 interacts with Toca-1 only in tubules.

To determine whether Cdc42 interaction was necessary for the formation of the Toca-1/N-WASP complex we used Cdc42 binding defective mutants, Toca-1MGD383-385IST and N-WASPH208D. Interestingly, N-WASPH208D was not competent to make a complex with Toca-1 suggesting that Cdc42 binding to N-WASP was essential for the formation of a trimer complex (Fig. 6B). For comparison, we also examined whether the N-WASPΔWA mutant formed a complex with Toca-1 and found that it did. However, the Toca-1/N-WASPΔWA complex had a null phenotype (Fig. 6C). The Toca-1 Cdc42 binding defective mutant MGD383-385IST was still able to complex with N-WASP and did not alter the phenotype of the complex dramatically (Fig. 6B).

### Cdc42 interaction with Toca-1 and N-WASP regulates membrane tubulation, vesicle formation and motility

**Figure 7:**
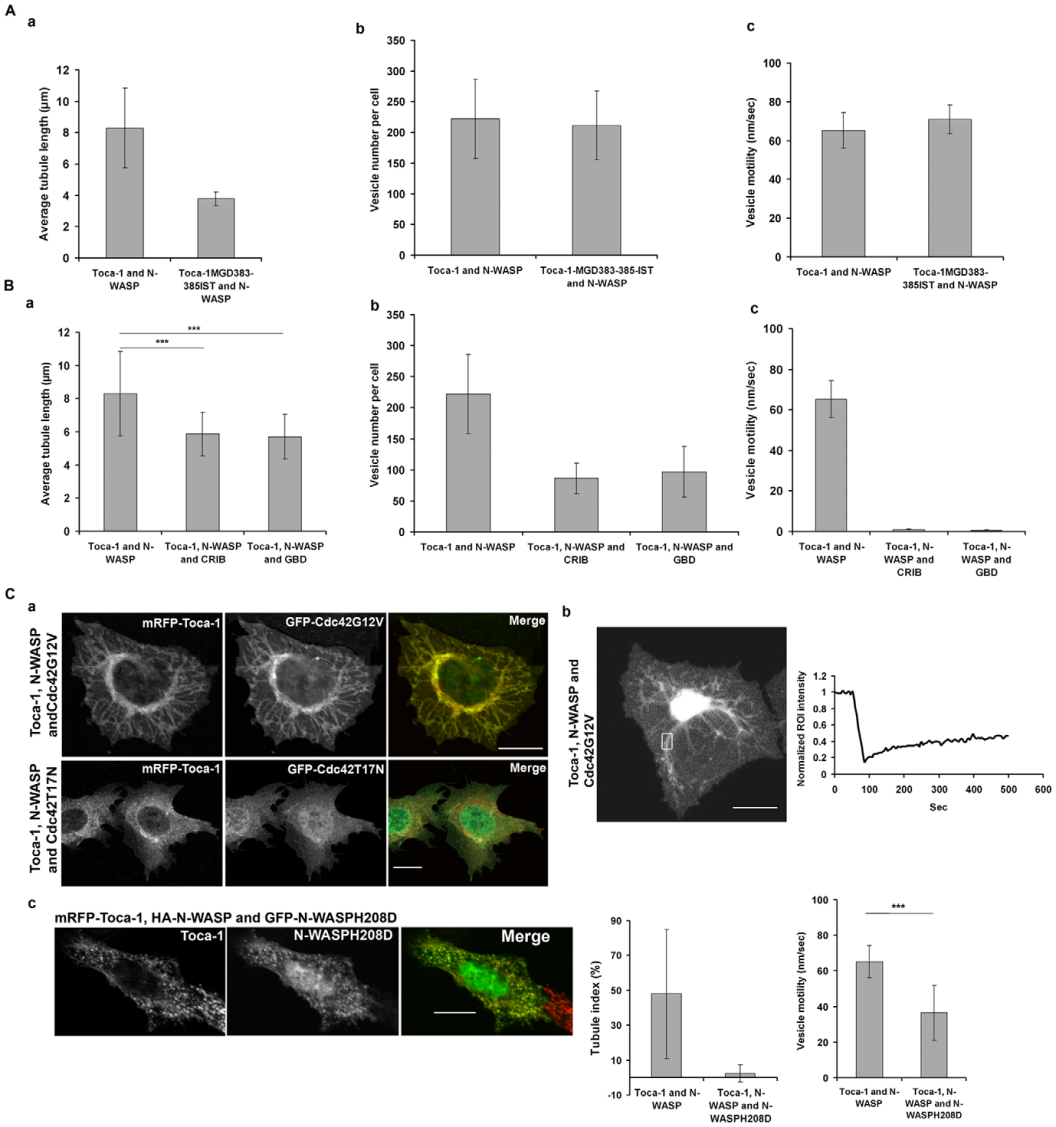
Effect of Toca-1MGD383-385IST and Cdc42 modulation on Toca-1/N-WASP phenotypes. (A). Cells were transfected with cDNA encoding the Cdc42 binding mutant of Toca-1MGD383-385IST with N-WASP as described in the Material and methods. Cell phenotypes of Toca-1 MGD383-385IST with N-WASP were analyzed for (a) tubulation, (b) vesicle number and (c) vesicle motility. (B). Cells were transfected with cDNA encoding Toca-1/N-WASP, CRIB or GBD domains and effects on (a) tubulation, (b) vesicle number or (c) vesicle motility. (C). Cells were transfected with Toca-1/N-WASP/Cdc42 combinations and allowed to express for 36 hours as described in the Material and methods. (a) Effect of Cdc42G12V or Cdc42T17N on the Toca-1/N-WASP phenotype. (b) FRAP analysis of the protein dynamics of Cdc42G12V/Toca-1/N-WASP transfected cells. (c) Effect of N-WASPH208D exchange into Toca-1/N-WASP induced structures. Left panel – Toca-1 image, middle panel – N-WASPH208D image, right panel – merge of the two images. Bar charts show statistical analysis of effects N-WASPH208D exchange into the Toca-1/N-WASP complex on tubulation and on vesicle motility. The methods for measuring tubule index and vesicle motility are described in the Material and methods section. Bar = 10 µm. For statistical analysis numbers are averages +/− S. D., with n = 6–10, from 2–3 experiments.

When the Cdc42 binding defective Toca-1MGD383-385IST mutant was expressed with N-WASP the length of tubules were dramatically shorter by approx 50% (Fig. 7A). In addition, vesicle size was reduced by the Toca-1 Cdc42 binding-defective mutant. These results suggest that direct binding of Cdc42 to Toca-1 regulates its F-BAR domain membrane tubulation activity. In contrast, when the N-WASPH208D mutant was used in combination with Toca-1 or Toca-1MGD383-385IST the tubule/vesicle phenotype was absent (Fig. 6B). Thus Cdc42 interaction with N-WASP is essential for complex formation and for tubule/vesicle formation.

Since it was not possible to use N-WASPH208D to examine the role of Cdc42-N-WASP interaction in membrane tubulation, vesicle formation or motility, we turned to other approaches. This included the use of proteins that bind to active Cdc42 and block its function GBD (GTPase binding domain) and CRIB (Cdc42 and Rac interacting binding site), dominant active (Cdc42G12V) and negative (Cdc42T17N) versions of Cdc42, respectively, and the exchange of N-WASPH208D into complexes of Toca-1/N-WASP. Cdc42 mutants (V12 and N17) and CRIB domains are widely used to study the function of this protein as well as other members of Rho family. It should be noted that the specificity of these proteins is not absolute. For instance, Cdc42N17 may target exchange factors that also activate Rac and Rho. The CRIB domain we use is derived from WASP and has high specificity for Cdc42, unlike the CRIB domain from PAK which binds Cdc42 and Rac equally well. Nevertheless, the WASP CRIB domain may have effects on Rac pathways at high concentration. In the present work, to address this specificity issue, we combine the use of Cdc42 mutants and CRIB protein with Cdc42 binding dead point mutations of N-WASP and Toca-1. CRIB and GBD are small 50–60 mer residue polypeptides derived from PAK and WASP proteins that bind to Cdc42-GTP specifically and inhibit signaling. Interestingly, both CRIB and GBD significantly reduced tubulation, vesicle formation and motility (Fig. 7B). Most dramatically affected by CRIB and GBD was vesicle motility. CRIB also affected the distribution of vesicles in cells as examined by confocal Z-sectioning of cells (Fig. S5). In control cells vesicles were found throughout the cell. In CRIB cells the number of vesicles were substantially reduced and only found in areas near the coverslip. We interpret these results to suggest that vesicle formation/motility requires Cdc42.

Next we used Cdc42G12V/T17N to explore the role of Cdc42-N-WASP interaction in the tubule/vesicle phenotype. Cells expressing Toca-1/N-WASP with Cdc42T17N had a null phenotype while most cells with Cdc42G12V possessed tubules but no vesicles (Fig. 7C, a). The tubules found in Cdc42G12V expressing cells were non-dynamic (Fig. 7C, b). To examine the role of Cdc42 interaction with N-WASP further we decided to try and exchange mutant GFP-N-WASPH208D into cells that had the Toca-1/N-WASP phenotype (Fig. 7C, c). We were able to follow the exchange by looking for GFP in the tubules/vesicles. GFP-N-WASPH208D inhibited tubule formation and vesicle motility (Fig. 7C, c). Taken together, these data suggest that Cdc42 interaction with N-WASP is a key event in the formation of the Cdc42-N-WASP-Toca-1 trimer complex which subsequently plays a role in three distinct steps of endocytosis; tubulation, vesicle formation and motility.

The above data suggest that Cdc42 may regulate endocytosis. To test this idea in a more physiological context we followed Rab5 membranes using GFP-Rab5. As shown in Table 3, the Rab5 vesicle motility was reduced in the presence of Cdc42G12V or Cdc42T17N, supporting a role for Cdc42 in early endosomal trafficking.

**Table 3:** Effect of Cdc42 on the motility of Rab5 vesicles.

Condition	Control	Cdc42G12V	Cdc42T17N
Rab5 vesicle motility (µm/sec)	0.108±0.077	0.072±0.043	0.072±0.036

CHO cells were transfected with GFP-Rab5 alone, GFP-Rab5 and Cdc42G12V, or GFP-Rab5 and Cdc42T17N. The timelapse acquisition was performed 24 hr post transfection. The motility of vesicles was tracked using metamorph software as described in Material and methods. For statistical analysis numbers are mean +/− S. D., with n = 6–10, from 2 experiments. Experimental data were analyzed by Student's t-test (p<0.001).

## Discussion

BAR domains were first identified in metazoan proteins BIN/Amphiphysin and yeast proteins (Rvs161/Rvs167; reduced viability upon starvation) prior to functional attributes of membrane remodeling being associated with the domain. Currently there are three distinct families of BAR domains; classical-BAR, F-BAR (e.g. Fes/CIP4) and I-BAR (e.g. IRSp53). Structural studies have revealed common elements of the BAR domain which includes dimerization modules that possess curvature and positively charged surfaces [11]. The role of BAR domains in membrane tubulation has opened-up the investigation of how membranes are remodeled during cellular processes. Recent work on the Toca family, FBP17, CIP4 and Toca-1, suggest a role for these proteins in endocytosis [13]–[15]. The presence of a SH3 domain in FBP17, CIP4 and Toca-1 that binds N-WASP provides a link to the actin machinery through the Arp2/3 complex. A third domain (HR1) of these proteins is the Cdc42 binding site. Unlike, FBP17 and CIP4, Toca-1 alone did not induce membrane tubulation. We found that expression of Toca-1 with N-WASP together was required to induce formation of membrane tubules and vesicles. The tubules and vesicles generated by Toca-1/N-WASP were dynamic, positive for Rab5, PIP_2_, and clathrin. Toca-1/N-WASP affected uptake of transferrin but not of cholera toxin B or of dextran. Previous work has shown that knockdown of all three Toca family proteins, FBP17, CIP4 and Toca-1, is required to inhibit endocytosis significantly [15] providing evidence for redundancy within the protein family. The colocalization marker and uptake data presented here suggest that the Toca-1/N-WASP complex is associated with clathrin-mediated endocytosis.

### Actin polymerization plays a role in membrane deformation and scission events

The N-WASP ΔWA mutant expressed with Toca-1 gave null phenotype suggesting that the N-WASP-Arp2/3 mediated actin polymerization plays an essential role in Toca-1-N-WASP induced tubule and vesicle formation. Data generated using Cyt. D also support a role for actin polymerization in membrane tubulation and vesicle formation - scission. Interestingly, an elegant study by Romer et al. showed that actin polymerization played an essential role in membrane scission events associated with membrane tubule invaginations generated by Shiga toxin [28].

### Membrane tubulation

We have shown that mutation of the F-BAR domain eliminates tubules and the N-WASP WA domain leads to a null phenotype. Thus the tubule phenotype we observe relies on the activity of these domains in combination and is therefore distinct from that observed with Shigella toxin [29]. FRET analysis revealed that Cdc42 interacted with N-WASP and Toca-1 on tubules. The Toca-1MGD383-385IST mutant is not able to bind Cdc42 and with N-WASP induced tubules that were dramatically shorter that wild-type protein. We interpret this data as evidence that Cdc42 interaction with Toca-1 regulates but is not essential for F-BAR domain activity. In contrast, the N-WASP mutants, H208D and ΔWA, expressed with Toca-1 did not give a phenotype. This result suggests that Cdc42 interaction with N-WASP, and N-WASP with Arp2/3, are essential for F-BAR domain activity and membrane remodeling. This is supported by the result obtained with exchange of N-WASP by N-WASPH208D which resulted in a reduced number of tubules. FRET data showed that the N-WASPH08D mutant did not form a complex with Toca-1 and this may be the reason for the null phenotype. In contrast, the N-WASPΔWA mutant did form a complex with Toca-1 but still had a null phenotype. From the protein-protein interaction-phenotype correlations we hypothesize that Cdc42-interaction with N-WASP is critical to “open” the N-WASP and expose the polyproline rich and WA domains, which then bind Toca-1 SH3 domain and the Arp2/3 complex, respectively. Binding of N-WASP polyproline rich domain by Toca-1 SH3 domain may reveal its F-BAR domain allowing it to couple membrane tubulation with actin polymerization via Arp2/3 complex.

### Vesicle formation

Since vesicle number was unaffected by the Toca-1MGD383-385IST mutation, Cdc42 interaction with Toca-1 is not important for vesicle formation. As the N-WASPH208D mutant had a null phenotype we could not use this mutant to investigate the role of Cdc42 interaction with N-WASP in vesicle formation or motility. To address the role of the Cdc42–N-WASP interaction in vesicle formation and motility we used a number of modulators of the Cdc42 pathway. Firstly, CRIB and GBD reduced vesicle number significantly and affected the distribution of vesicles within the cell. Secondly, in cells expressing high levels of Cdc42G12V or Cdc42T17N with Toca-1/N-WASP no vesicles were seen. This result suggests that cycling of active/inactive Cdc42 is important for N-WASP function in vesicle formation.

### Vesicle motility

FRET analysis revealed that Cdc42 interacted with N-WASP but not Toca-1 on vesicles. The vesicles generated by Toca-1/N-WASP were motile and possessed an actin tail in the shape of a comet reminiscent of bacterial/viral motility in mammalian cells. Toca-1-dynamin coexpression generated non-motile vesicles suggesting N-WASP was critical for motility. Toca-1MGD383-385IST or the F-BAR domain mutants did not affect vesicle motility significantly. In contrast, the addition of either GBD or CRIB to Toca-1/N-WASP cells eliminated vesicle motility. Lastly, exchange of N-WASPH208D into vesicles inhibited their motility. CRIB domain microinjection into cells immediately inhibited vesicle motility (data not shown). Thus Cdc42 interaction with N-WASP and not Toca-1 is essential for vesicle motility. As found *in vitro*, Toca-1 interaction with N-WASP may be required to activate actin polymerization *in vivo*, to drive vesicle motility. Further work is necessary to investigate the role of Toca-1 in controlling N-WASP actin polymerization activity, *in vivo*, during vesicle movement.

The results presented here show that Cdc42 regulates the coupling of BAR domain activity with actin dynamics as a means to remodel membranes may represent a general mechanism used in the formation of structures such as filopodia, podosomes, T-tubules and membrane vesicles [11]. There are close similarities between the filopodia generating ability of the I-BAR domain protein IRSp53 [22], [30], [31] and the membrane vesicle generating ability of Toca-1. Both have Cdc42 binding sites and N-WASP binding sites. In the case of IRSp53 mediated filopodia formation its SH3 domain interacts with a number of proteins involved in actin dynamics; Mena [32] Eps8 [33] as well as N-WASP [22]. For Toca-1, its SH3 domain seems to be specific for N-WASP but there is no reason why other proteins involved in actin dynamics could not bind it.

### Implications for endocytosis

Work on CIP4 and FBP17 suggests that these proteins play a role in endocytosis that is redundant with Toca-1 [15]. During the preparation of this manuscript, Fricke et. al., [34] reported the role of the Drosophila CIP4 homolog in morphological pathways associated with bristle formation. Their phenotypic observations with regards to membrane tubules and vesicles are largely similar to those presented here. In the present study we have focused on the role of Cdc42 in formation of a trimer complex with Toca-1/N-WASP and regulation of its activity. Our protein-protein interaction with phenotype correlations suggests that Cdc42 interaction with N-WASP is a critical first step to the formation of the Cdc42-N-WASP-Toca-1 trimer complex. The function of this trimer complex is to couple F-BAR domain activity with actin polymerization to give dynamic membrane tubulation and the formation of motile vesicles. Cdc42-Toca-1 interactions affect tubule length and therefore F-BAR domain activity. Thus Cdc42 activity may regulate endocytic trafficking pathways by controlling the formation and activity of N-WASP-Toca-1 complex.

## Supporting Information

Table S1 Mutants and descriptions. Mutants' name, location of the mutations and functions.
(0.04 MB DOC)

Figure S1 Toca-1/N-WASP induces tubules and vesicles in Hela, N1E115 and COS7 cells. mRFP-Toca-1 and GFP-N-WASP were transfected into Hela cells (A), N1E115 cells (B) and COS7 cells (C). Confocal images were shown in the sequence of mRFP-Toca-1, GFP-N-WASP and merged. Vesicles are shown in a-c in panel A, d-f in panel B and a-c in Panel C. Tubules are shown in d-i in Panel A, a-c in Panel B and a-c in Panel C. Bar = 10 µm.
(4.04 MB TIF)

Figure S2 Tubules induced by Toca-1 and N-WASP colocalised with PMT. Cells were transfected with YFP-PMT (A) or YFP-PMT together with mRFP-Toca-1 and HA-N-WASP (B) and left to express for 36 hr as described in the Material and methods section. The cells were examined by confocal microscopy. The images in panel B were shown in the sequence of PMT, Toca-1 and merge. The zoomed images of the boxed area were shown below respective image. Bar = 10 µm.
(2.62 MB TIF)

Figure S3 Vesicles or tubules induced by Toca-1 and N-WASP colocalize with Rab5, Rab7, clathrin, actin and PH domain probes, but not caveolin. (A) CHO cells were transfected with mRFP-Toca-1 and HA-N-WASP followed with antibody (Rab5, Rab7 or Lamp-1) staining. Vesicles or tubules induced by Toca-1 and N-WASP were analyzed for colocalization by intensity tracing through the vesicle/tubules. First two panels following intensity tracing are the actual vesicles/tubules that were examined. The schematic of the vesicles (third panel) shows the intensity line. Intensity analysis was carried out as described under “Materials and Methods.” Intensity tracings in other panels (B–E) are done similarly as that of panel A. Bar = 10 µm (B) CHO cells transfected with mRFP-Toca-1, HA-N-WASP and GFP-actin were fixed and examined with confocal microscopy. The Upper panel shows the cell with vesicles and the lower panel shows the cell with tubules. left panel-Toca-1, middle panel-actin, right panel-merge. Bar = 10 µm (C) CHO cells transfected with mRFP-Toca-1 and GFP-clathrin (a), or mRFP-Toca-1, HA-N-WASP and GFP-clathrin (b and c) were fixed and examined with confocal microscopy. left panel -Toca-1, middle panel-clathrin, right panel-merge. Bar = 10 µm (D) CHO cells transfected with Myc-Toca-1, GFP-N-WASP and RFP-caveolin were fixed and examined with confocal microscopy. left panel -N-WASP, middle panel-caveolin, right panel-merge. Bar = 10 µm (E) CHO cells were transfected with mRFP-Toca-1, HA-N-WASP and CFP-PLCδ-PH (a), mRFP-Toca-1, HA-N-WASP and CFP-Akt-PH (b and c), mRFP-Toca-1, HA-N-WASP and GFP-BTK-PH (d and e). The cells were fixed and imaged with confocal microscopy 24 hrs after transfection. left panel-Toca-1, middle panel-individual PH domain probe, right panel-merge. Bar = 10 µm.
(2.40 MB TIF)

Figure S4 Uptake assays of endocytic markers by Toca-1 and N-WASP expressing cells. Cells were transfected with Toca-1 and N-WASP and left to express the proteins for 24–36 hr. Markers for uptake pathways were then added and uptake monitored for 10 min for transferrin (A and B) and 30 min for dextran and cholera toxin B (C). The cells were then washed, fixed and examined by confocal microscopy. Bar = 10 µm.
(2.81 MB TIF)

Figure S5 Effect of CRIB domain on Toca-1/N-WASP phenotype. Cells were transfected with mRFP-Toca-1 and GFP-N-WASP cDNA with and without CRIB cDNA and left to express mRFP/GFP for 36 hr as described in the Material and methods section. Cells were then selected based on Toca-1 mRFP fluorescence intensity and scored for (A) tubule length and (B) vesicle number per cell. In addition, (C) for each cell a Z-stack of approx 28 sections (0.3 µm per section) was acquired and vesicle number per section measured. In (A) and (B) each point represents an individual cell. In (C) the vesicle number is an average +/− S. D., with n = 4 from 2–3 experiments.
(0.36 MB TIF)

Movie S1 Toca-1/N-WASP vesicle dynamics. CHO cells were transfected with cDNA encoding mRFP-Toca-1 and GFP-N-WASP. After 24 hr, the time-lapse acquisition was performed on an inverted Olympus FV1000 confocal microscope at 10 sec interval for 4 min. Image acquisition in the mRFP and GFP channels was done sequentially. Bar = 10 µm.
(4.20 MB MOV)

Movie S2 mRFP-Toca-1/GFP-N-WASP induced tubule/vesicle transitions with TIRFM. CHO cells were transfected with cDNA encoding mRFP-Toca-1 and GFP-N-WASP. After 24 hr, the time-lapse acquisition was performed on an inverted Olympus FV1000 TIRF microscope at 10 sec interval for 60 min. The penetration depth was approx 100 nm from the coverslip. Image acquisition in the mRFP and GFP channels was taken sequentially. Bar = 10 µm.
(5.22 MB MOV)

Movie S3 mRFP-Toca-1/HA-N-WASP/GFP-actin induced vesicle motility. CHO cells were transfected with cDNA encoding mRFP-Toca-1, HA-N-WASP and GFP-actin. After 24 hr, the time-lapse acquisition was performed on an inverted Olympus FV1000 confocal microscope at 2.3 sec interval for ∼2 min. Image acquisition in the mRFP and GFP channels was done sequentially. Bar = 10 µm.
(10.07 MB MOV)
