# Supplementary material for: Cdc42 Interaction with N-WASP and Toca-1 Regulates Membrane Tubulation, Vesicle Formation and Vesicle Motility: Implications for Endocytosis
Source: PLoS One. 2010 Aug 13;5(8):e12153. doi: 10.1371/journal.pone.0012153 (PMC2921345; doi:10.1371/journal.pone.0012153)
Supplement: Table S1 — Mutants and descriptions. Mutants' name, location of the mutations and functions. (0.04 MB DOC) [file pone.0012153.s001.doc]

| Mutant name | Location of the mutations | function |
| --- | --- | --- |
| Myc-Toca-1W518K | SH3 domain | Defective in SH3 domain |
| mRFP-Toca-1W518K | SH3 domain | Defective in SH3 domain |
| mRFP-Toca-1K33QR35Q | F-BAR domain | Defective in lipid binding and membrane deformation |
| mRFP-Toca-1K51QK52Q | F-BAR domain | Defective in lipid binding and membrane deformation |
| mRFP-Toca-1R112QK113Q | F-BAR domain | Defective in lipid binding and membrane deformation |
| mRFP-Toca-1MGD383-385IST | HR1 domain | Defective in Cdc42 binding |
| GFP-N-WASPH208D | CRIB domain | Defective in Cdc42 binding |
| GFP-N-WASPΔWA | WA domain | Defective in Arp2/3 activation |
| Dynamin-K44A | Nucleotide binding site | Defective in GTP binding |
| Cdc42G12V | GTPase domain | Defective in GTPase activity |
| Cdc42T17N | Nucleotide binding site | Defective in binding affinity to GTP/GDP |
