# Supplementary figures and images for: Cdc42 Interaction with N-WASP and Toca-1 Regulates Membrane Tubulation, Vesicle Formation and Vesicle Motility: Implications for Endocytosis
Source: PLoS One. 2010 Aug 13;5(8):e12153. doi: 10.1371/journal.pone.0012153 (PMC2921345; doi:10.1371/journal.pone.0012153)

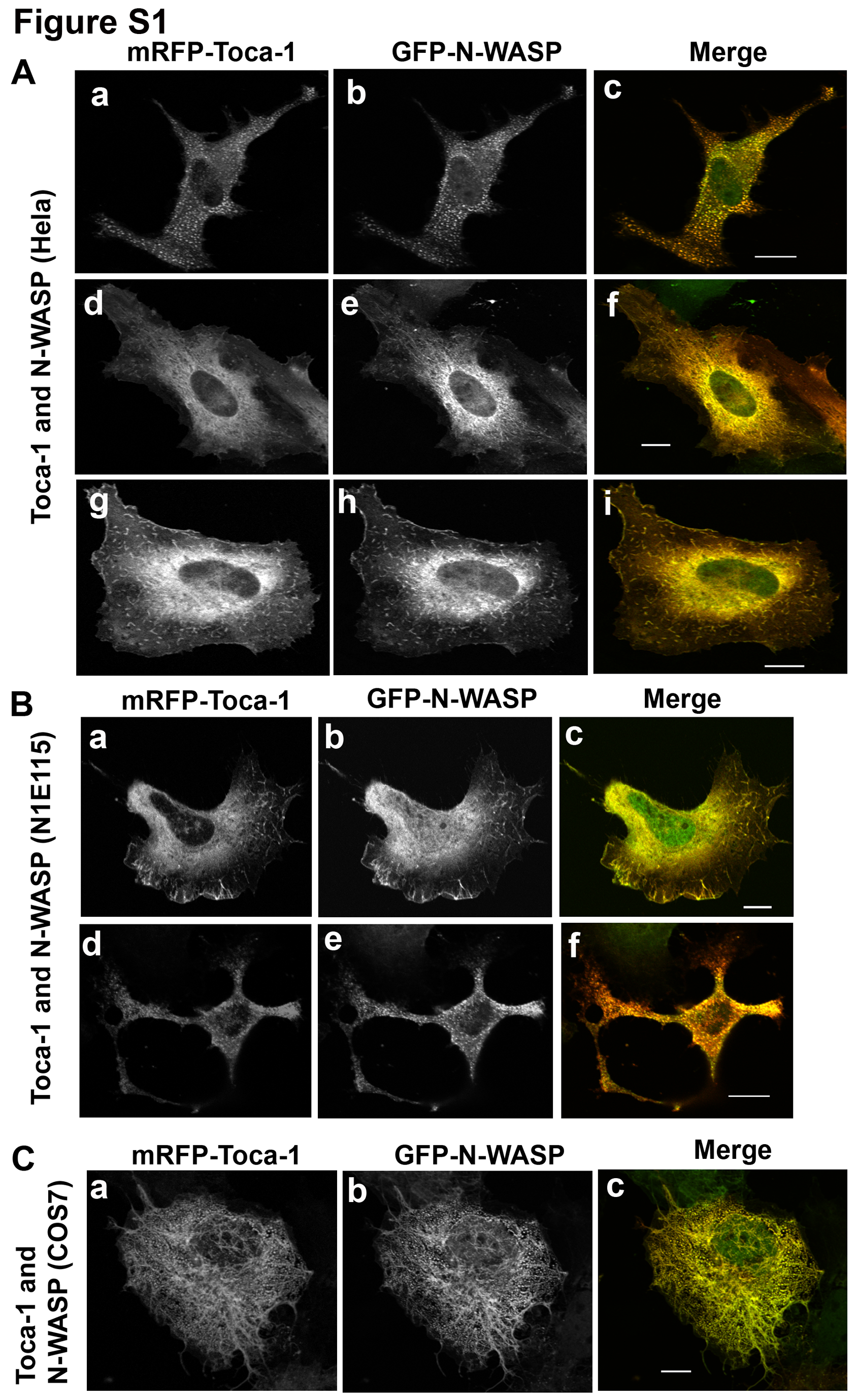

Supplement: Figure S1 — Toca-1/N-WASP induces tubules and vesicles in Hela, N1E115 and COS7 cells. mRFP-Toca-1 and GFP-N-WASP were transfected into Hela cells (A), N1E115 cells (B) and COS7 cells (C). Confocal images were shown in the sequence of mRFP-Toca-1, GFP-N-WASP and merged. Vesicles are shown in a-c in panel A, d-f in panel B and a-c in Panel C. Tubules are shown in d-i in Panel A, a-c in Panel B and a-c in Panel C. Bar = 10 µm. (4.04 MB TIF) [file pone.0012153.s002.tif]

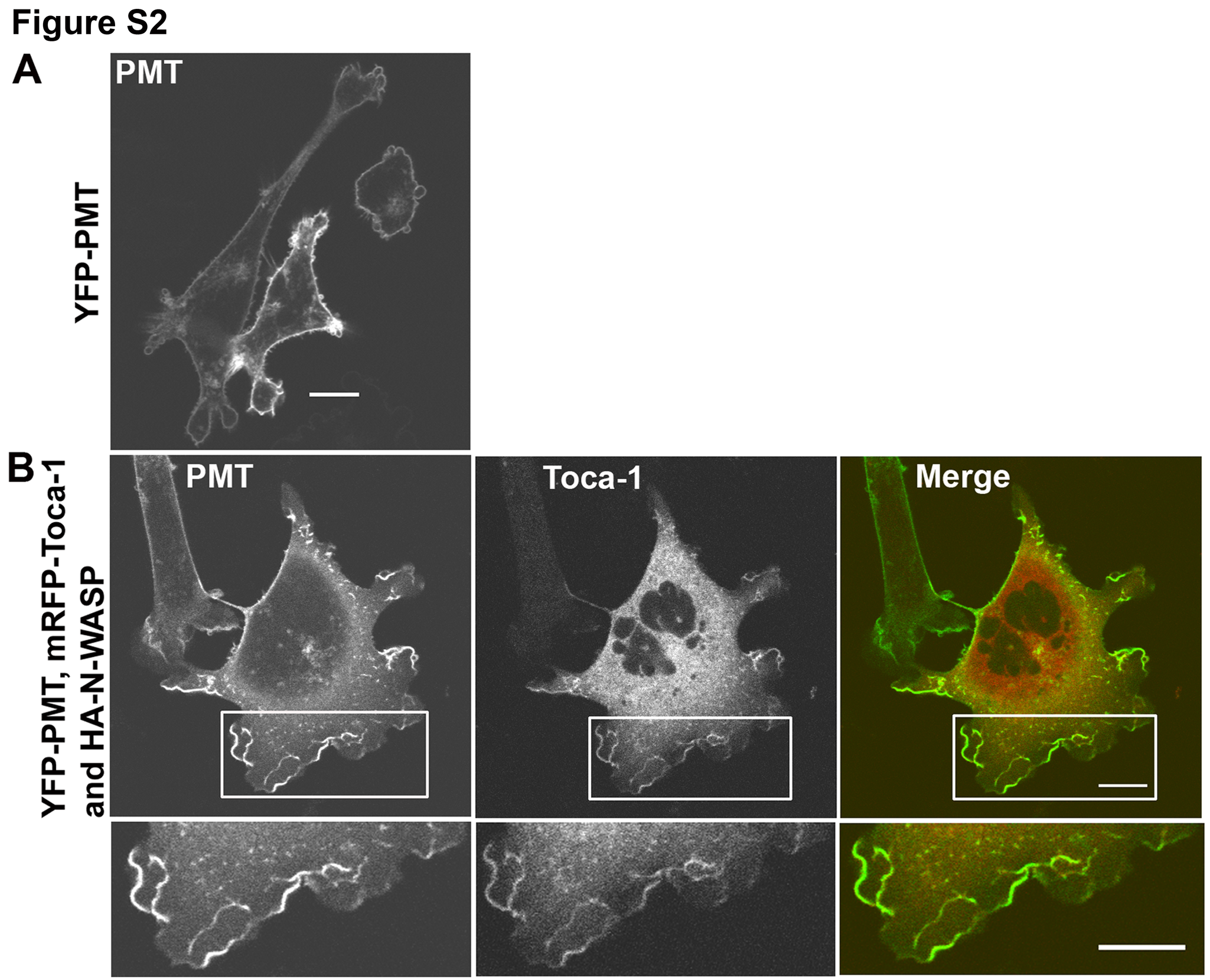

Supplement: Figure S2 — Tubules induced by Toca-1 and N-WASP colocalised with PMT. Cells were transfected with YFP-PMT (A) or YFP-PMT together with mRFP-Toca-1 and HA-N-WASP (B) and left to express for 36 hr as described in the Material and methods section. The cells were examined by confocal microscopy. The images in panel B were shown in the sequence of PMT, Toca-1 and merge. The zoomed images of the boxed area were shown below respective image. Bar = 10 µm. (2.62 MB TIF) [file pone.0012153.s003.tif]

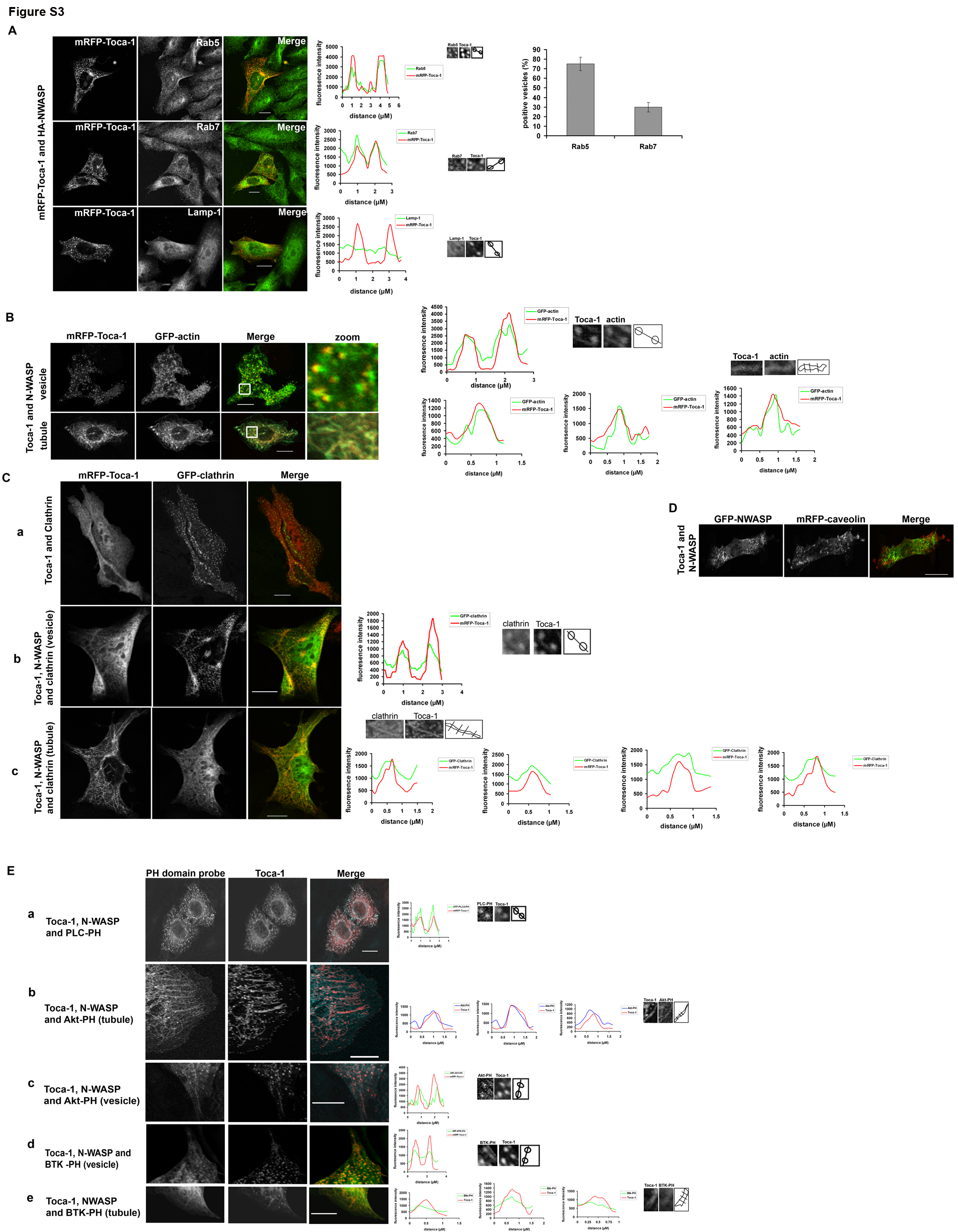

Supplement: Figure S3 — Vesicles or tubules induced by Toca-1 and N-WASP colocalize with Rab5, Rab7, clathrin, actin and PH domain probes, but not caveolin. (A) CHO cells were transfected with mRFP-Toca-1 and HA-N-WASP followed with antibody (Rab5, Rab7 or Lamp-1) staining. Vesicles or tubules induced by Toca-1 and N-WASP were analyzed for colocalization by intensity tracing through the vesicle/tubules. First two panels following intensity tracing are the actual vesicles/tubules that were examined. The schematic of the vesicles (third panel) shows the intensity line. Intensity analysis was carried out as described under “Materials and Methods.” Intensity tracings in other panels (B–E) are done similarly as that of panel A. Bar = 10 µm (B) CHO cells transfected with mRFP-Toca-1, HA-N-WASP and GFP-actin were fixed and examined with confocal microscopy. The Upper panel shows the cell with vesicles and the lower panel shows the cell with tubules. left panel-Toca-1, middle panel-actin, right panel-merge. Bar = 10 µm (C) CHO cells transfected with mRFP-Toca-1 and GFP-clathrin (a), or mRFP-Toca-1, HA-N-WASP and GFP-clathrin (b and c) were fixed and examined with confocal microscopy. left panel -Toca-1, middle panel-clathrin, right panel-merge. Bar = 10 µm (D) CHO cells transfected with Myc-Toca-1, GFP-N-WASP and RFP-caveolin were fixed and examined with confocal microscopy. left panel -N-WASP, middle panel-caveolin, right panel-merge. Bar = 10 µm (E) CHO cells were transfected with mRFP-Toca-1, HA-N-WASP and CFP-PLCδ-PH (a), mRFP-Toca-1, HA-N-WASP and CFP-Akt-PH (b and c), mRFP-Toca-1, HA-N-WASP and GFP-BTK-PH (d and e). The cells were fixed and imaged with confocal microscopy 24 hrs after transfection. left panel-Toca-1, middle panel-individual PH domain probe, right panel-merge. Bar = 10 µm. (2.40 MB TIF) [file pone.0012153.s004.tif]

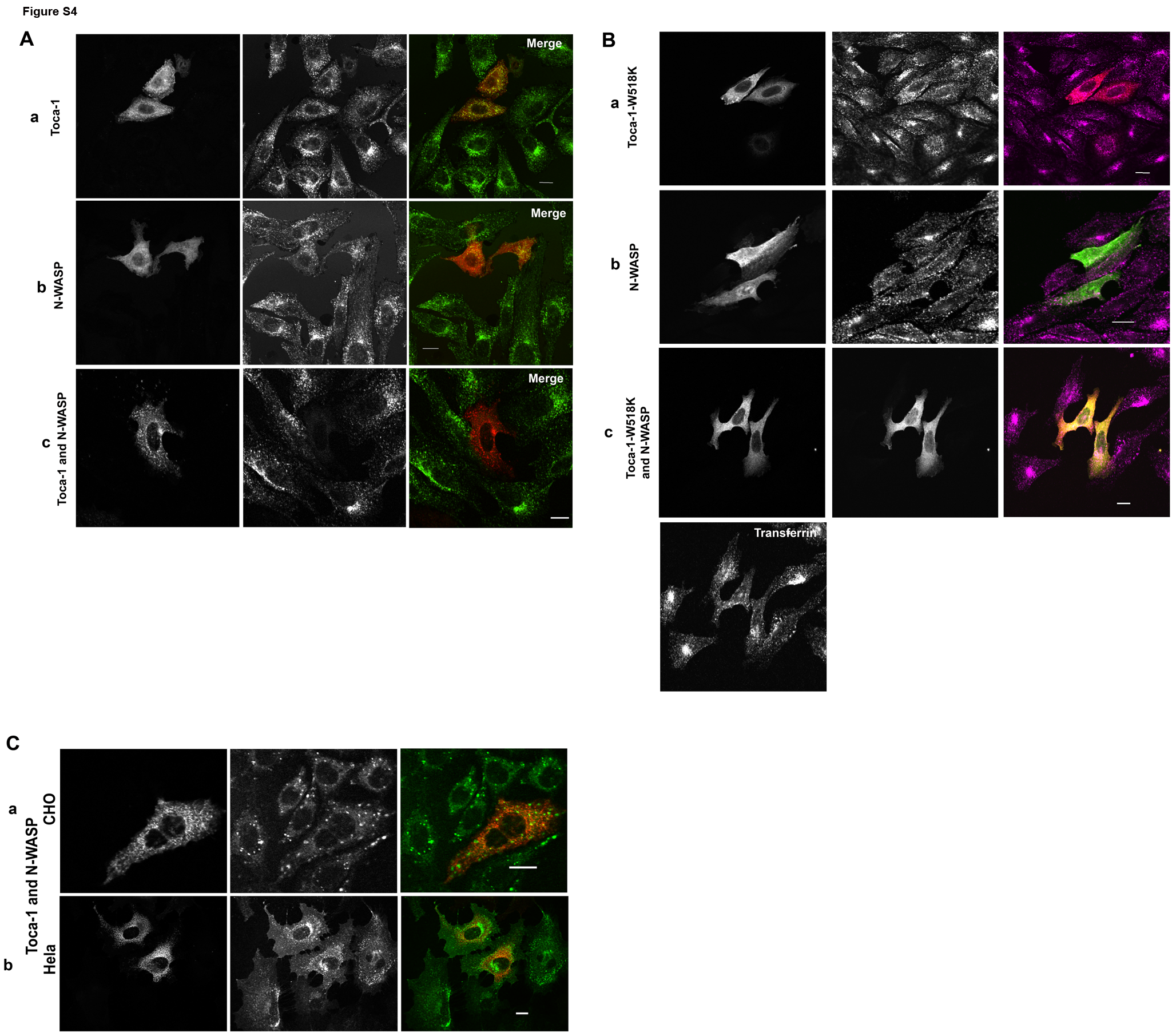

Supplement: Figure S4 — Uptake assays of endocytic markers by Toca-1 and N-WASP expressing cells. Cells were transfected with Toca-1 and N-WASP and left to express the proteins for 24–36 hr. Markers for uptake pathways were then added and uptake monitored for 10 min for transferrin (A and B) and 30 min for dextran and cholera toxin B (C). The cells were then washed, fixed and examined by confocal microscopy. Bar = 10 µm. (2.81 MB TIF) [file pone.0012153.s005.tif]

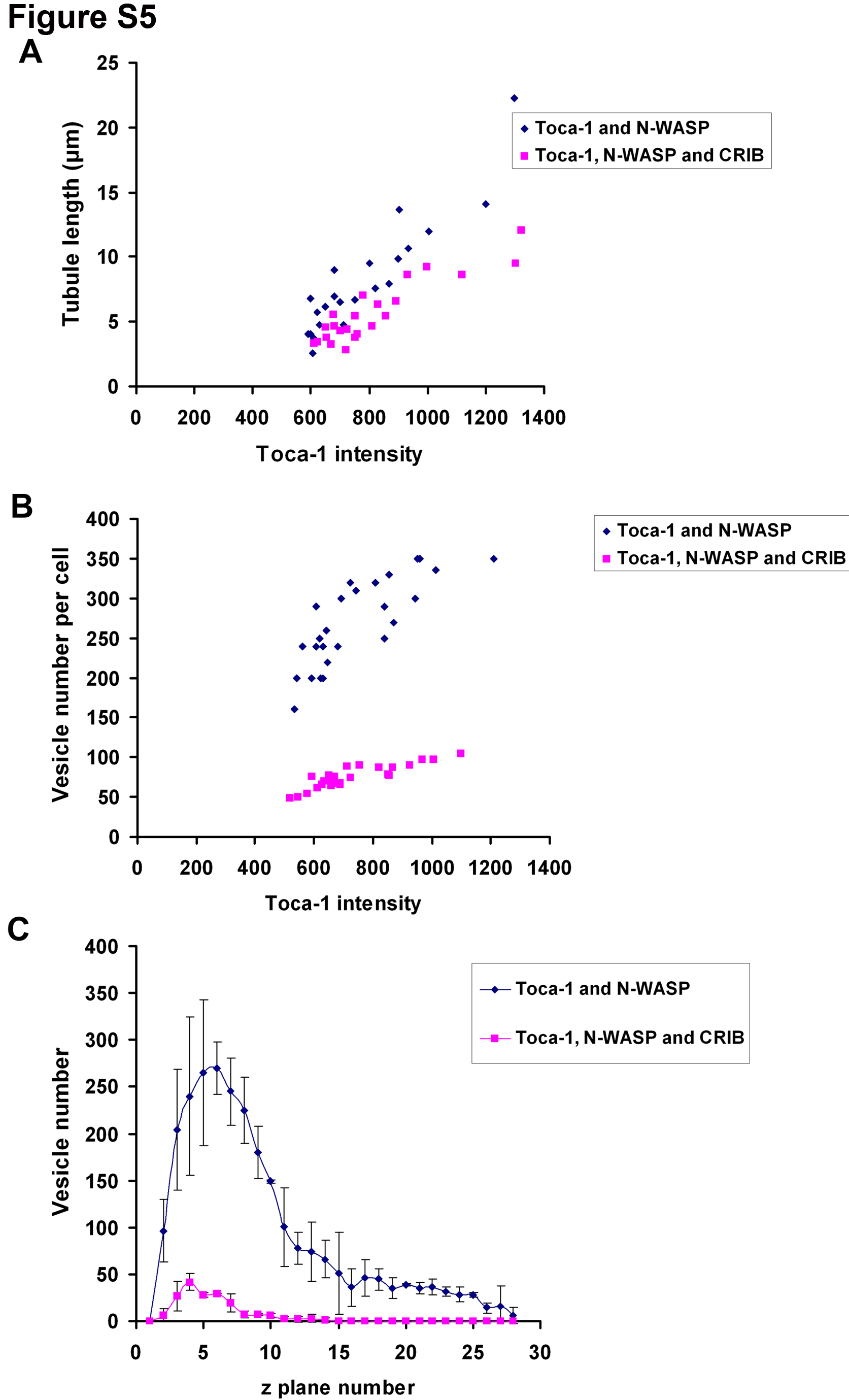

Supplement: Figure S5 — Effect of CRIB domain on Toca-1/N-WASP phenotype. Cells were transfected with mRFP-Toca-1 and GFP-N-WASP cDNA with and without CRIB cDNA and left to express mRFP/GFP for 36 hr as described in the Material and methods section. Cells were then selected based on Toca-1 mRFP fluorescence intensity and scored for (A) tubule length and (B) vesicle number per cell. In addition, (C) for each cell a Z-stack of approx 28 sections (0.3 µm per section) was acquired and vesicle number per section measured. In (A) and (B) each point represents an individual cell. In (C) the vesicle number is an average +/− S. D., with n = 4 from 2–3 experiments. (0.36 MB TIF) [file pone.0012153.s006.tif]
